# Exploring Aromatic Cage Flexibility Using Cosolvent Molecular Dynamics Simulations—An In-Silico Case Study of Tudor Domains

**DOI:** 10.1021/acs.jcim.4c00298

**Published:** 2024-05-21

**Authors:** Christopher Vorreiter, Dina Robaa, Wolfgang Sippl

**Affiliations:** Department of Medicinal Chemistry, Institute of Pharmacy, Martin-Luther-University of Halle-Wittenberg, 06120 Halle, Saale, Germany

## Abstract

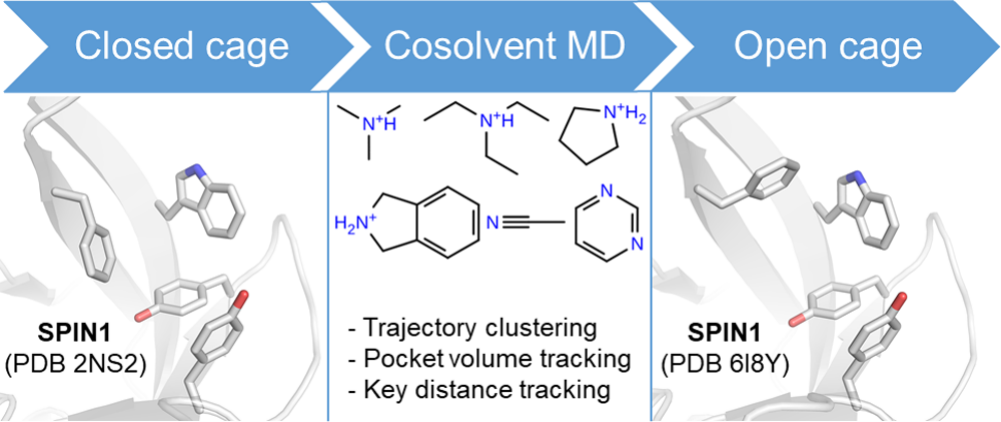

Cosolvent molecular dynamics (MD) simulations have proven to be powerful in silico tools to predict hotspots for binding regions on protein surfaces. In the current study, the method was adapted and applied to two Tudor domain-containing proteins, namely Spindlin1 (SPIN1) and survival motor neuron protein (SMN). Tudor domains are characterized by so-called aromatic cages that recognize methylated lysine residues of protein targets. In the study, the conformational transitions from closed to open aromatic cage conformations were investigated by performing MD simulations with cosolvents using six different probe molecules. It is shown that a trajectory clustering approach in combination with volume and atomic distance tracking allows a reasonable discrimination between open and closed aromatic cage conformations and the docking of inhibitors yields very good reproducibility with crystal structures. Cosolvent MDs are suitable to capture the flexibility of aromatic cages and thus represent a promising tool for the optimization of inhibitors.

## Introduction

The family of Tudor domains is a known group of methyl-lysine and methyl-arginine reader proteins with the ability to recognize respective post-translational modifications. Typically, Tudor domains appear either as single domains or in tandems and show barrel-like protein structures. Within this protein family, the recognition of methylated lysine or arginine residues occurs with the aid of so-called aromatic cages. These pockets consist of several aromatic amino acid residues, which are able to accommodate positively charged ammonium moieties such as methylated lysine and arginine residues.^1,2^ Overexpression of tudor domain-containing proteins have been linked to several severe diseases, and hence represent potential drug targets.^3−11^ Small molecules targeting these reader proteins usually act by occupying the aromatic cage, thereby competing with the binding of the methylated lysine or arginine residues.^12,13^ In order to enable structure-based approaches for inhibitor design, a thorough investigation of the conformational arrangement and flexibility of the aromatic cage residues is crucial. Indeed, resolved crystal structures of several Tudor domains have revealed that the aromatic cage can adopt multiple conformations in the presence of different ligands as well as in apoform.^14−17^

Cosolvent molecular dynamics (MD) simulations have been shown to be useful in-silico tools for analyzing protein surfaces in terms of binding sites and hotspots.^18,19^ Compared to classical MD simulations using water as a single solvent, this approach is characterized by blending organic molecules into the aqueous phase. Depending on the nature and characteristics of the used cosolvents, specific interactions with the protein surface are observable in respective trajectories. Various approaches have already been presented, differing in simulation times, utilized sets of probe molecules, cosolvent concentrations, result analysis, investigated proteins, and other parameters.^18−22^ Other works focused on the exploration of different types of binding sites, for example, allosteric and cryptic pockets, or mapping hotspots for protein–protein interactions.^23−26^

Conformational changes upon ligand binding (induced fit) can hamper the accurate binding mode prediction using classical docking studies.^27^ Classical MD simulations and clustering can be performed to better predict the binding mode, however using pure water as solvent can only yield restricted insight since the chemical properties of ligands are not taken into consideration.^28^ In this study, we wanted to investigate whether the use of cosolvent MDs can not only help recognize the hotspots, but also sample the binding site conformations, specifically binding sites comprising aromatic cages. In this work, we focused on two Tudor domain containing proteins namely Spindlin 1 (SPIN1) and survival motor neuron protein (SMN).

**Figure 1:**
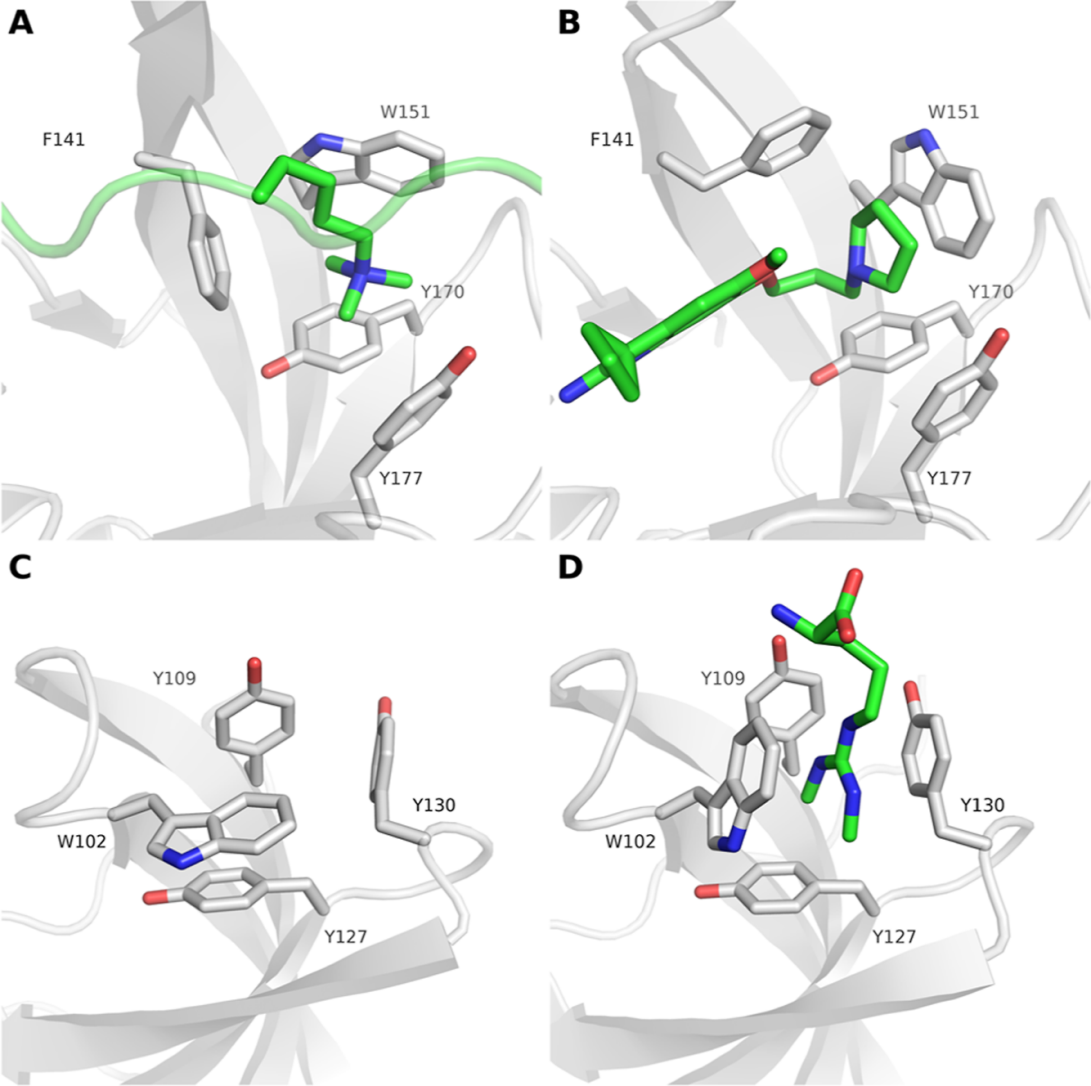
Aromatic cages found in SPIN1 and SMN adopt multiple conformations. (A) Closed state of the aromatic cage in SPIN1 domain 2 in complex with H3K4me3 (PDB ID 4MZG). (B) Open state of the aromatic cage in SPIN1 domain 2 in complex with inhibitor A366 (PDB ID 6I8Y). (C) Closed state of the aromatic cage found in the apo protein structure (PDB ID 1MHN) of the SMN Tudor domain. (D) Open state of the aromatic cage of the SMN Tudor domain in complex with dimethylated arginine (PDB ID 4A4E).

SPIN1 is a reader protein that features three Tudor domains. It is known to bind specific histone marks containing methylated lysines (H3K4me3, H4K20me3) as well as asymmetrically dimethylated arginine moieties like H3R8me2a.^29−33^ Crystallographic studies revealed that both domains 1 and 2 are potential targets for small molecule inhibitors as well. Monovalent binding modes involving domain 1 and bivalent binding modes additionally addressing domain 2 have been described.^13,34,35^ Crystal structures of SPIN1 also revealed that the aromatic cage can adopt different conformations. For example, while the apoform and peptide-bound form in domain 2 show a closed cage conformation^31,36^ (Figure 1A), inhibitor-bound forms majorly show an open cage conformation^13^ (Figure 1B). Our previous studies on this protein showed that rigid docking is not successful in reproducing the binding mode of the potent inhibitor A366 using the closed cage conformation as a docking template. In contrast, induced fit docking was discovered to be a more suitable tool to address the necessary protein flexibility. However, prestudies with MD simulations were needed to identify the most flexible residues, so that they could be manually assigned in the induced fit docking protocol. Longer MD-simulations in water (500 ns) were not successful in capturing the inhibitor-bound conformation of the aromatic cage.^37^

SMN comprises a single Tudor domain which binds arginine- and glycine-rich elements of Sm proteins that are associated with snRNPs (small nuclear ribonucleoprotein particles).^38,39^ Other protein targets like RNA polymerase II^40^ and Coilin^41^ have been described as well. All targets usually carry posttranslational modifications showing symmetrically dimethylated arginines (Rme2s). The aromatic cage found in the SMN Tudor domain was discovered to specifically bind these modified residues.^40,42−45^ Besides, it has been shown that asymmetrically dimethylated arginines (Rme2a) are also recognized by the aromatic pocket.^46^ Liu et al. presented small molecule probes that are able to occupy the binding site as well.^12^ While inspecting the available crystal structures of the SMN Tudor domain, multiple aromatic cage conformations are detectable depending on the presence and properties of the bound ligand (Figure 1C,D).

In this study, we set to extend the application of cosolvent MDs from mere binding site mapping and investigate whether they can represent—when using properly chosen cosolvents—a rational and computationally less expensive methodology to sample binding site conformations with focus on aromatic cages.

## Materials and Methods

### Protein Preparation

The following preparation steps were carried out using the graphical user interface of Schrödinger’s Maestro program.^47^ All protein structures were retrieved from the Protein Data Bank (PDB; http://www.rcsb.org).^48^ Water and buffer molecules were initially deleted. The Protein Preparation Wizard^49,50^ was used afterward to carry out the following steps: bond orders were assigned and hydrogen atoms were added. The option to fill in missing side chains and loops, if required, was enabled. The proteins’ termini were capped at the end of the process. If cocrystallized ligands were part of the structure, protonation states were generated using Epik (pH 7 ± 2). Subsequently, the hydrogen bond network was automatically optimized at pH 7.0. In the last preparation step, the OPLS 2005 force field was used to perform final energy minimization.^51−54^

### MD Simulations

The Amber 22 software package was used to carry out all MD simulations.^55^ Initial protein coordinates were obtained from the prepared apo protein structures. At first, the protein chains were prepared using pdb4amber and parametrized according to the ff14SB force field.^56,57^ AM1-BCC atomic charges were assigned to the cosolvent molecules that were subsequently parametrized using the General Amber Force Field 2.^58−60^ TLeap was used to set up the final system in the environment of a binary solvent mixture. The probe molecules were initially distributed as a layer around the protein surface. In a second layer, TIP3P^61^ water molecules were added to achieve a final probe concentration of ∼1 M (Table S1). The resulting truncated octahedral periodic box was neutralized using either sodium or chloride ions, depending on the protein and probe molecules used. For isoindoline, it was observed that the repulsive forces among the positively charged molecules were not sufficient in order to achieve equal probe distribution. Pi–pi-stacking interactions between the probe molecules prevented a homogeneous distribution. Therefore, a dummy atom was placed in the center of the aromatic ring and was parametrized by a Lennard-Jones potential using *R*_min_ = 12 Å and ε = 0.0001 kcal/mol as parameters. Homogenous probe distribution in the aqueous phase was verified by radial distribution functions (Figure S1). Each system initially underwent two minimization steps, a heating step and a pressure equilibration step. The first minimization stage included 1000 iterations of steepest descent affecting the whole system. The following conjugate gradient minimization, including 2000 iterations, affected only solvent molecules. Afterward, the system was heated to production temperature (300 K) through 100 ps of MD simulation. During this process, the protein chains were restrained with a force constant of 10 kcal mol^–1^ Å^–2^. Constant volume periodic boundary was set to equilibrate the temperature of the system by Langevin thermostat using a collision frequency of 2 ps^–1^. After reaching the target temperature, a pressure equilibration step was executed for 100 ps while applying a constant pressure of 1 bar. Subsequently, 10 replicates of classical MD simulations, each of 20 ns length, were carried out for every system using different, randomly assigned seeds, respectively. The first 10 ns of simulation were considered an additional equilibration phase in which the probe molecules got the opportunity to evenly mix with the water molecules. The last 10 ns represented the actual production stage in which the aromatic cage conformations were investigated. These production stages were collected and concatenated into a single trajectory for each probe molecule containing 10,000 frames, respectively. All simulations took place at a constant temperature of 300 K using Langevin thermostat with a collision frequency of 2 ps^–1^, the time step was set to 2 fs. During all temperature equilibrations and actual MD simulations, the Particle Mesh Ewald method was applied, which featured a nonbonded cutoff distance of 10.0 Å for long-range electrostatic interactions.^62^ Additionally, the SHAKE algorithm was enabled to constrain all bonds involving hydrogens.^63^

### Trajectory Analysis

Trajectory clustering as well as distance, RMSD and RMSF calculations were performed using CPPTRAJ which is a part of Amber Tools.^64^ All trajectories were prealigned on the first frame, considering the C_α_ atoms of the respective aromatic cage residues. The hierarchical agglomerative clustering algorithm was chosen to carry out the clustering approach. The clustering process was run multiple times to get a series of clusters in which the number of output clusters was varied from two to 20. For each clustering run, success parameters like the Davies–Bouldin index, the pseudo F-statistic (pSF) and the SSR/SST ratio (sum of squares regression/total sum of squares) were calculated. These parameters assisted in the choice of a reasonable cluster count.^65^ Additionally, the representative cluster structures were inspected visually in order to evaluate the quality of clustering. As a distance metric for clustering, the RMSD values of the cage residues’ side chains (heavy atoms) were used.

### Pocket Volume Analysis

Fpocket^66^ was used to determine the pocket shapes for selected X-ray structures showing open cage conformations (Figure S2). If multiple reference structures with open pockets were selected, the extracted shapes were superimposed. MDpocket^67^ was used as a tool to analyze protein cavities in the dynamic structures obtained from the MD simulations. Using the shape input generated by fpocket, pocket descriptors like volume metrics could be calculated for every input frame. For the sake of comparability, MDpocket was also used to calculate the binding pocket volume values of the reference X-ray structures (Table 1).

**Table 1:** Measured Atom Distances and Calculated Pocket Volumes for the Aromatic Cages in Selected Reference X-Ray Structures

protein	parameter	closed state	open state 1	open state 2
SPIN1 domain 1	PDB ID	2NS2	6I8Y	4H75
	atom distance [Å] (W72-C_H2_–F251-C_G_)	6.0	9.5	8.9
	pocket volume [Å^3^]	0	315	316
SPIN1 domain 2	PDB ID	2NS2	6I8Y	
	atom distance [Å] (F141-C_Z_–Y177-C_G_)	6.7	10.5	
	pocket volume [Å^3^]	183	397	
SMN Tudor domain	PDB ID	1MHN	4QQ6	7W2P
	atom distance [Å] (W102-C_H2_–Y130-C_G_)	4.8	7.0	7.6
	pocket volume [Å^3^]	0	157	211

### Analysis of Representative Cluster Structures

Schrödinger’s Maestro was used to visualize and further analyze the clustering output obtained from the trajectory analysis.^47^ RMSD values for the representative cluster structures were calculated using the Superposition panel. These values were determined considering the side chain coordinates (heavy atoms) of the aromatic cage residues. In advance, all structures were prealigned on the cage residues‘ C_α_ atoms of the reference structure. The atom distances of the key aromatic residues were also measured using the graphical user interface of Maestro.

### Docking Study

Schrödinger’s LigPrep was used to prepare the inhibitor structures.^68^ Standard settings were kept, including Epik for the generation of ionization states and OPLS 2005 was chosen as the utilized force field.^69−71^ For SPIN1 inhibitor A366, 50 ligand conformers were sampled for the docking input using ConfGen.^72,73^ Prior to the actual docking process, receptor grids were generated by defining the grid center with the aid of the aromatic cage residues. The side length of the midpoint cube was set to 8 Å, the outer box featured additional 15 Å of side length for ligand placement. Docking was carried out using Glide in standard precision mode.^74−77^ The standard docking protocol was complemented by enabling the setting to reward intramolecular hydrogen bonds and the setting to include input ring conformations. An output of one docking pose per input conformer was chosen, resulting in 50 output poses for the SPIN1 inhibitor A366. For all dockings in the SMN Tudor domain, the number of output poses was set up to 50 as only one conformer per ligand (due to the lack of rotatable bonds) served as docking input.

### Plot Generation and Molecular Visualization

The violin plots, which visualize the volume and distance distributions, were created using Matplotlib.^78^ All remaining plots that are shown in this study were created using Microsoft Excel. The figures visualizing extracted trajectory frames as well as docking poses and experimentally determined binding poses were produced utilizing PyMol.^79^ 2D inhibitor and probe molecule depictions were exported from Marvin Sketch^80^ and Schrödinger’s Maestro.^47^

## Results and Discussion

**Figure 2:**
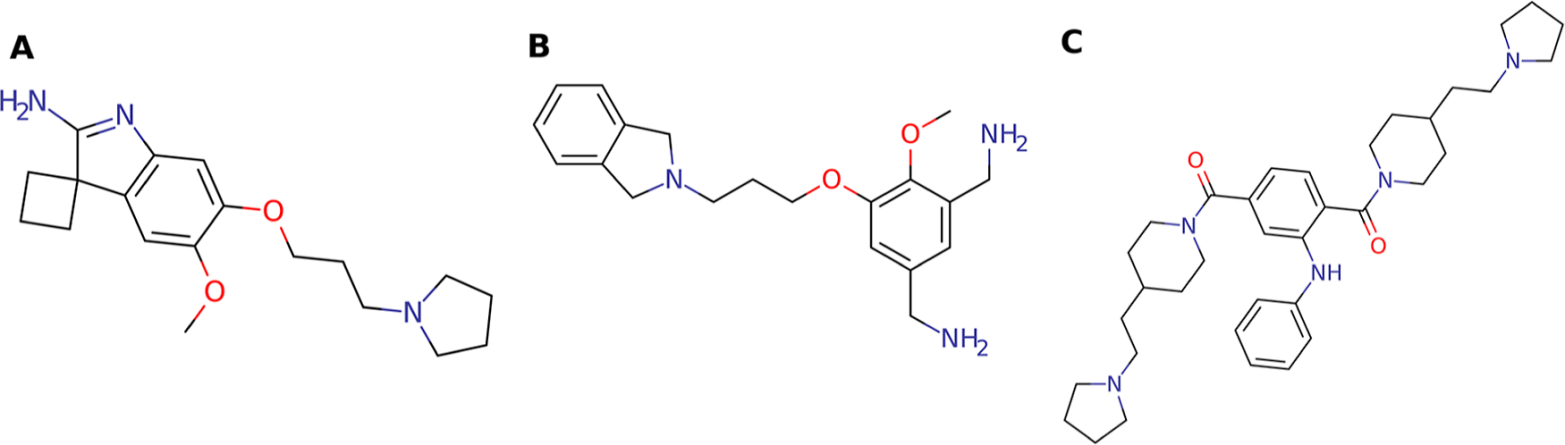
SPIN1 inhibitor structures: A366 (A), MS-31 (B), and EML405 (C).

**Figure 3:**
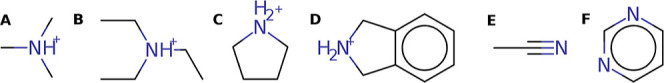
Chemical structures of the used cosolvent molecules in their respective protonation states: trimethylamine (A), triethylamine (B), pyrrolidine (C), isoindoline (D), acetonitrile (E), and pyrimidine (F).

To simulate the binding of inhibitors to aromatic cages, we focused on water-miscible cosolvents that can mimic protonated amines or guanidines. To this end, two probe molecules were chosen which are commonly found in SPIN1 inhibitors (Figure 2), namely pyrrolidine and isoindoline in protonated form (Figure 3C,D). Furthermore, the protonated forms of trimethylamine and triethylamine (Figure 3A,B) were used since these plain substructures are also known to be common aromatic cage binders.^81,82^ To investigate whether the presence of a positively charged amino group in the cosolvent is necessary to address and sample the conformation of the aromatic cage, two additional neutral cosolvents, namely acetonitrile and pyrimidine (Figure 3E,F), were investigated. It is worth noting that the latter cosolvents still comprise a polarizable nitrogen atom.

The apo protein structures of SPIN1 (PDB ID 2NS2) and SMN Tudor (PDB ID 1MHN) defined the initial coordinates for all simulations. At first, the proteins were covered in a layer of probe molecules. In order to achieve the desired cosolvent concentration of 1 M, water molecules were added accordingly (Table S1). The resulting systems were simulated 10 times for 20 ns. The last 10 ns of every MD simulation were considered for the conformational analysis of the aromatic cages. To this end, corresponding trajectory frames were concatenated so that a single trajectory of 100 ns per protein and probe molecule was obtained.

To assess the stability of the proteins during the MD simulation, root-mean-square deviations (RMSD) and fluctuations (RMSF) of the heavy atom coordinates were computed. The latter were compared with the reported *B*-factors in the respective crystal structures. It is observable that all RMSD values remain mostly below 3 Å and stabilize throughout the simulations (Figure S3). The RMSF plots suggest protein stability as well and exhibit similar patterns to the respective *B*-factor plot, both showing some regions with high fluctuations that correspond to flexible loop structures (Figure S4).

Initially, we retrieved all available PDB structures of the investigated proteins and analyzed the different experimentally solved aromatic cage conformations. To this end, RMSD matrices were generated by comparing the RMSD values of the aromatic cage residues in all available protein structures in order to allow the identification of clusters of similar pocket conformations. For every detected cluster, a “reference X-ray structure” was chosen representing the conformational state of the whole cluster. Generally, the states of the aromatic pockets could be differentiated in open and closed conformations.

Further analysis of the obtained MD trajectories was carried out using multiple approaches. Clustering of the MD trajectories was performed to obtain representative cluster structures. In order to detect the conformational state of the aromatic cage, i.e. open or closed cage conformation, the representative cluster structures were compared to the selected reference X-ray structures by calculating RMSD values of the respective aromatic cage residues. Additionally, the pocket volumes as well as distances between key aromatic residues over the MD simulation time were calculated in order to track conformational changes in the aromatic cage.

**Figure 4:**
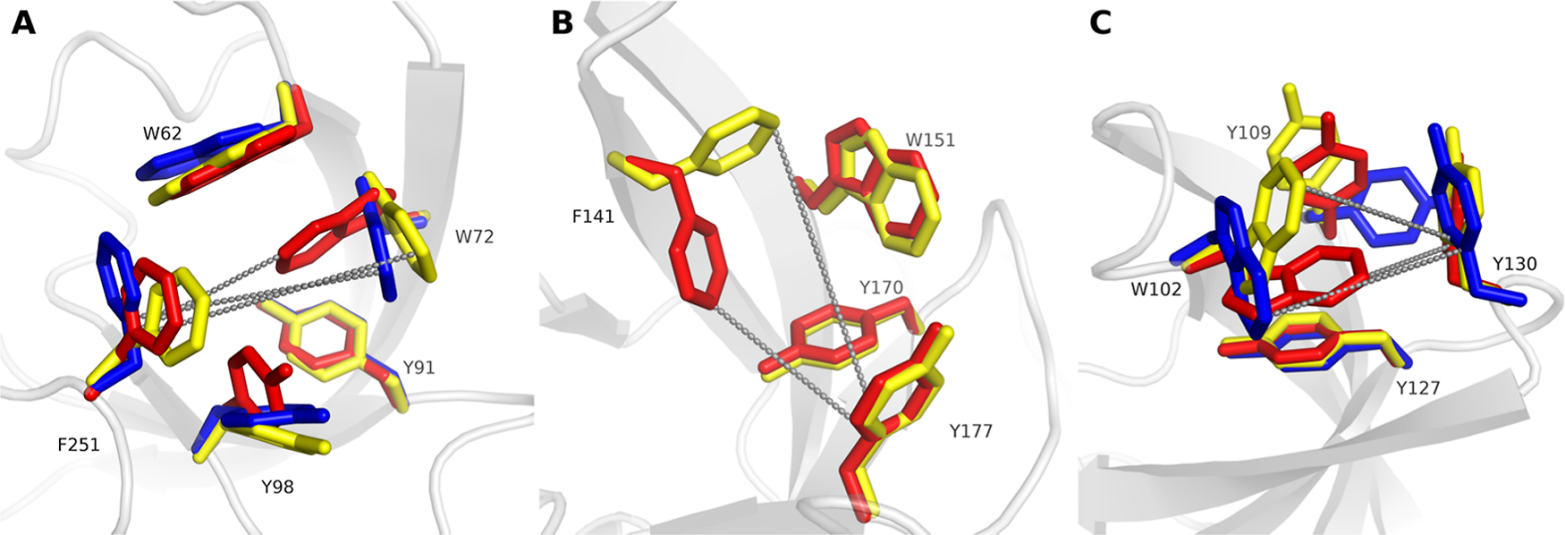
Selected reference X-ray structures visualizing the conformational flexibility of the investigated aromatic pockets. Distances between key aromatic residues are visualized by dashed lines. (A) SPIN1 domain 1—the closed state (PDB ID 2NS2) is depicted with red sticks, the first open state (PDB ID6I8Y) with yellow sticks and the second open state (PDB ID 4H75) with blue sticks. (B) SPIN1 domain 2—the closed state (PDB ID 2NS2) is depicted with red sticks, the open state (PDB ID 6I8Y) with yellow sticks. (C) SMN Tudor—the closed state (PDB ID 1MHN) is depicted with red sticks, the first open state (PDB ID4QQ6) with yellow sticks and the second open state (PDB ID 7W2P) with blue sticks.

The atom pairs for distance measuring were selected assuming that the aromatic binding site residues are either flexible or rather rigid upon ligand binding. Hence, crystal structures showing open and closed aromatic cages were first compared in order to assess the flexibility of all aromatic cage residues. Subsequently, the distance between the most flexible residue and the oppositely located residue was calculated as a measure of cage conformation (Figure 4).

### SPIN1 Domain 1

**Figure 5:**
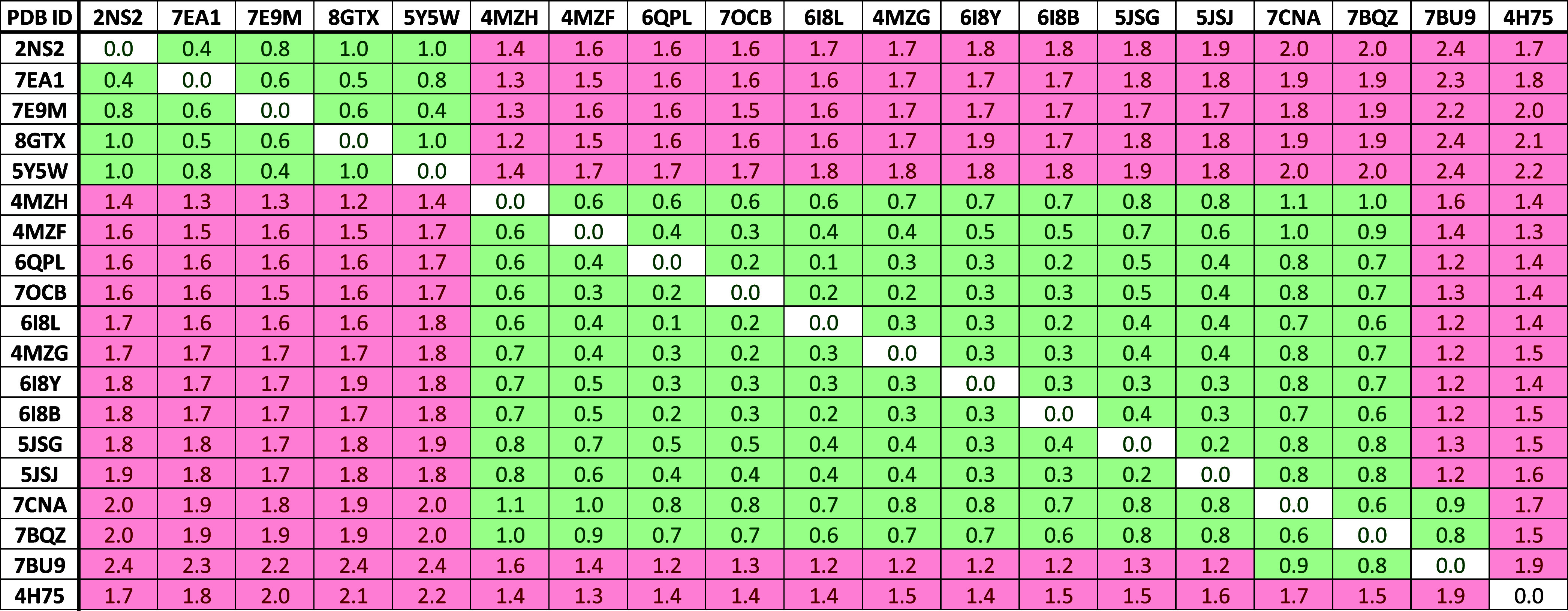
RMSD matrix of available SPIN1 crystal structures. The values were calculated for the aromatic cage residues of domain 1. A threshold of 1.2 Å was applied to highlight clusters of similar cage conformations.

The aromatic cage found in domain 1 of SPIN1 is formed by five amino acid residues: W62, W72, Y91, Y98 and F251 (Figure 4A). To date, 19 crystal structures of SPIN1 are retrievable on the PDB. In order to identify the different aromatic cage conformations among these structures, all structures were superposed and the RMSD values of the above-mentioned cage residues were calculated considering the coordinates of the side chains’ heavy atoms. As evident in the RMSD matrix (Figure 5), two clusters could be identified. The first cluster comprises structures that show a closed aromatic cage conformation, with residue W72 hampering the accessibility to the binding site. The second cluster, showing open-cage conformation, mainly contains structures in which fragments, (bivalent) inhibitors, and methylated arginine/lysine moieties are cocrystallized. The open conformation is characterized by the flipping-out of the aromatic cage residues W72 and Y98 (Figure 4A). In this work, we selected the apo protein structure (PDB ID 2NS2) and the structure of SPIN1 cocrystallized with inhibitor A366 (PDB ID 6I8Y) to serve as reference X-ray structures for both closed and open cage conformations, respectively (Figure 4A). Additionally, PDB ID 4H75 was chosen as a third reference X-ray structure since it could not be assigned to any cluster. The structure also shows an open-cage conformation; however the side chain of F251 shows a significant difference in its orientation when compared to other open-cage structures (Figure 4A).

**Figure 6:**
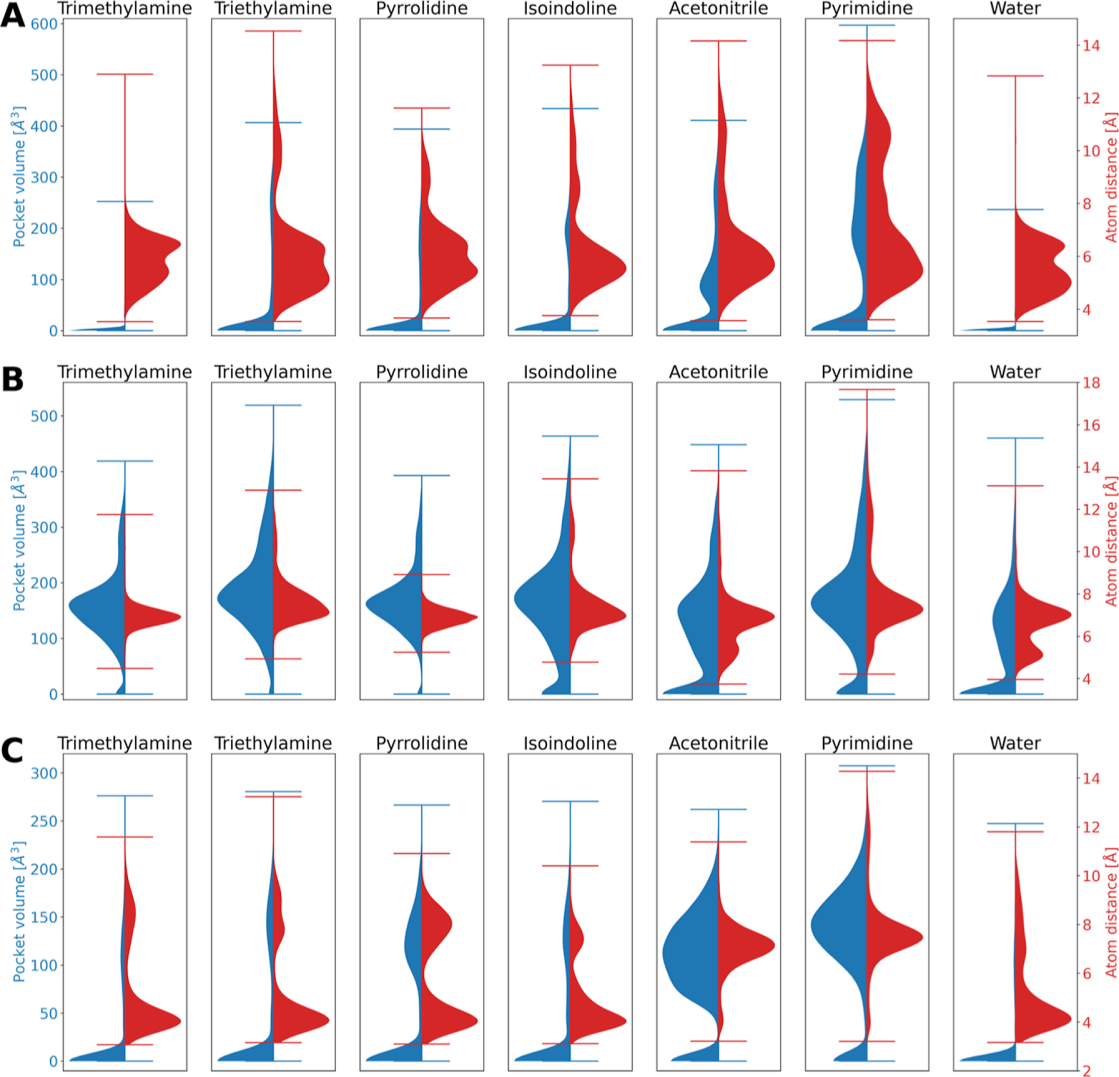
Pocket volume (blue) and atom distance (red) distributions for the aromatic cage of SPIN1 domain 1 (A), SPIN1 domain 2 (B) and the SMN Tudor domain (C) throughout the MD simulations performed in the presence of different probe molecules.

In order to investigate whether the open and closed forms of the aromatic cage in domain 1 of SPIN1 could be successfully sampled using the different cosolvents, we first tracked the pocket volumes as well as the distance between predefined key aromatic residues over the MD simulations. Previous studies have already shown that calculating the distances between the aromatic amino acid residues in cage structures can differentiate between open and closed cages.^83^ Here, we additionally used pocket volume calculations executed by MDpocket to further differentiate between both cage forms. Visual analysis of domain 1 in available crystal structures revealed that the distance between W72-C_H2_ and F251-C_G_ shows significant difference between open (9.5/8.9 Å) and closed (6.0 Å) cage conformations (Figure 4A and Table 1) and can thus be used to differentiate between the conformational states. The density distributions for the distance values and pocket volumes that were traced during the simulations were visualized with the aid of violin plots (Figure 6A).

The atom distance distributions determined for the simulations using trimethylamine and pure water reveal distance values majorly lying between 4 and 8 Å, suggesting that the aromatic residues did not undergo any significant movement and that the aromatic cage is mainly remaining in the initial closed conformation. Meanwhile, during the simulations using triethylamine, pyrrolidine, isoindoline, acetonitrile, and pyrimidine, we could observe distance values reaching or even exceeding the values measured for the open cage conformations. Especially in the case of the simulations using pyrimidine as probe molecule, the shift of the density distribution to higher distance values is well observable.

Similar observations could be made upon the pocket volume calculations. Initially, pocket volumes of 0 Å^3^ (closed cage; PDB ID 2NS2), 315 Å^3^ (open cage; PDB ID 6I8Y) and 316 Å^3^ (open cage; PDB ID 4H75) were calculated for the reference X-ray structures. The pocket volume tracking on the obtained MD trajectories of simulations performed in the presence of trimethylamine and pure water confirm that the aromatic cage remained in a closed conformation with the pocket volume of 0 Å^3^. Meanwhile, the other probe molecules allowed (slight) shifts to higher pocket volumes with the most significant volume increase observed when pyrimidine was used as a probe molecule.

Subsequently, all obtained trajectories were clustered based on the RMSD of the aromatic cage residues of domain 1 (W62, W72, Y91, Y98, F251) using a hierarchical agglomerative clustering approach, and representative structures of each cluster were further analyzed. To assess the presence of the open cage conformations in the representative cluster structures, the heavy-atom RMSD of the aforementioned residues’ side chains with respect to the corresponding residues in the reference structures was calculated. Additionally, pocket volumes and distances between F251-C_G_ and W72-C_H2_ were measured and averaged for the entire cluster. In Table 2, all clustering results are listed.

**Table 2:**
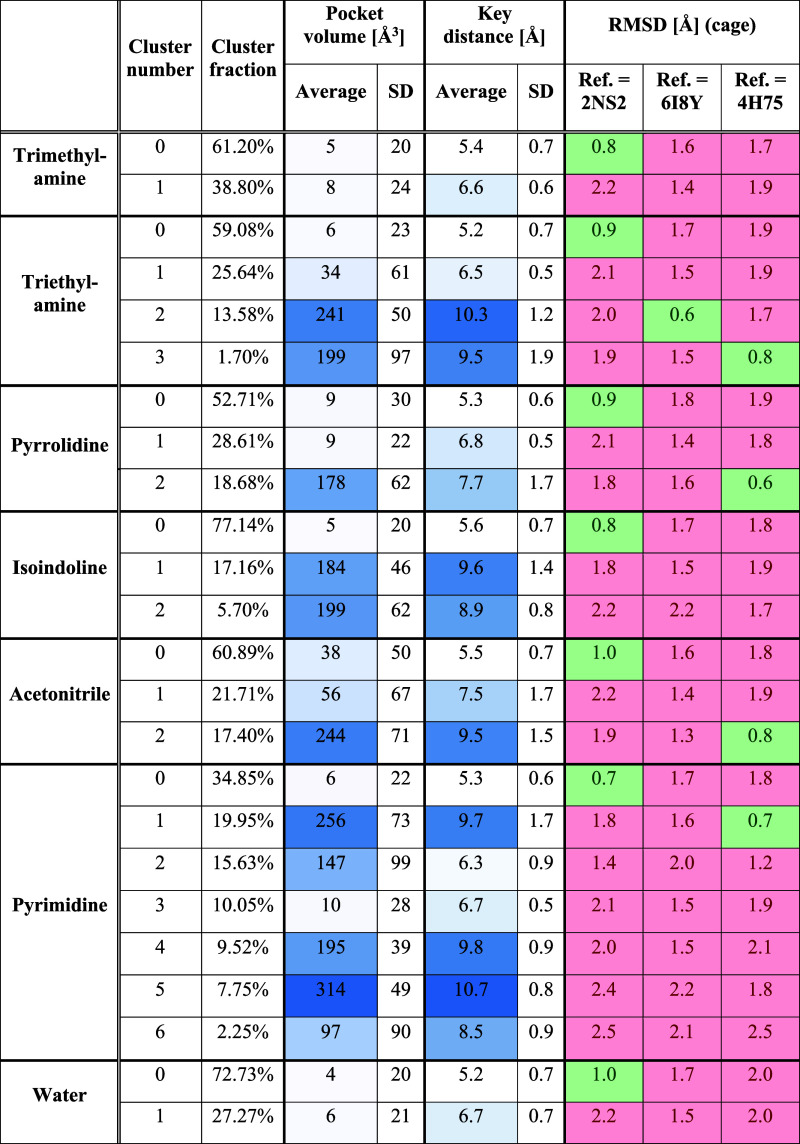
Clustering Results for the Aromatic Cage Found in SPIN1 Domain 1 and Calculated Descriptors for the Obtained Clusters (Volumes and Distances) and Representative Cluster Structures (RMSD)

**Figure 7:**
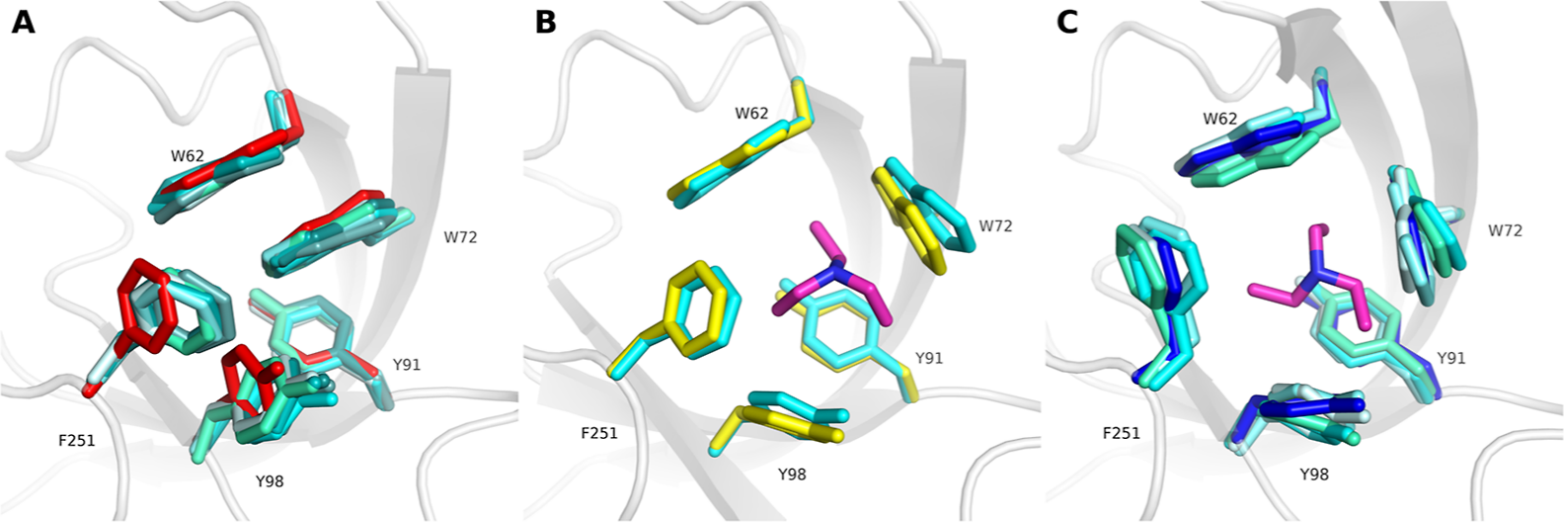
Cosolvent MDs are capable of sampling the aromatic cage conformations found in selected reference X-ray structures for SPIN1 domain 1. Triethylamine probe molecules are exemplarily shown with purple sticks. (A) The closed cage conformation of 2NS2 is shown with red sticks, the highest populated cluster structures using all probe molecules show similar conformations (depicted in different shades of cyan). (B) The open state of 6I8Y is shown with yellow sticks, triethylamine as a probe molecule was capable of sampling this conformation (cluster 2, shown with cyan-colored sticks). (C) The reference state of 4H75 is shown with dark blue sticks; simulations with triethylamine (cluster 3), pyrrolidine (cluster 2), acetonitrile (cluster 2) and pyrimidine (cluster 1) delivered similar conformations (shown in different shades of cyan).

We considered a conformation to be successfully sampled if an RMSD value below 1.2 Å was achieved. This equals the threshold applied in the RMSD matrix for assigning the available crystal structures to structural clusters (Figure 5). Analyzing the trajectory clustering results revealed that the highest populated clusters of all MD simulations adopt a closed-cage conformation, as demonstrated by the low RMSD values with respect to the apo protein structure (PDB ID 2NS2). This confirms the observation that has already been made while analyzing the pocket volume and key distance distributions: the closed conformation is the preferred state in all simulations (Figure 7A). Considering the RMSD values calculated with respect to the first open-cage reference X-ray structure (PDB ID 6I8Y), the respective cage conformation could only be reproduced using triethylamine as a probe molecule (Figure 7B). In contrast, the open state represented by the PDB structure 4H75 was sampled by multiple probe molecules (Figure 7C), namely triethylamine, pyrrolidine, acetonitrile, and pyrimidine. The simulations in pure water and in mixture with trimethylamine did not yield any open cage structures.

**Figure 8:**
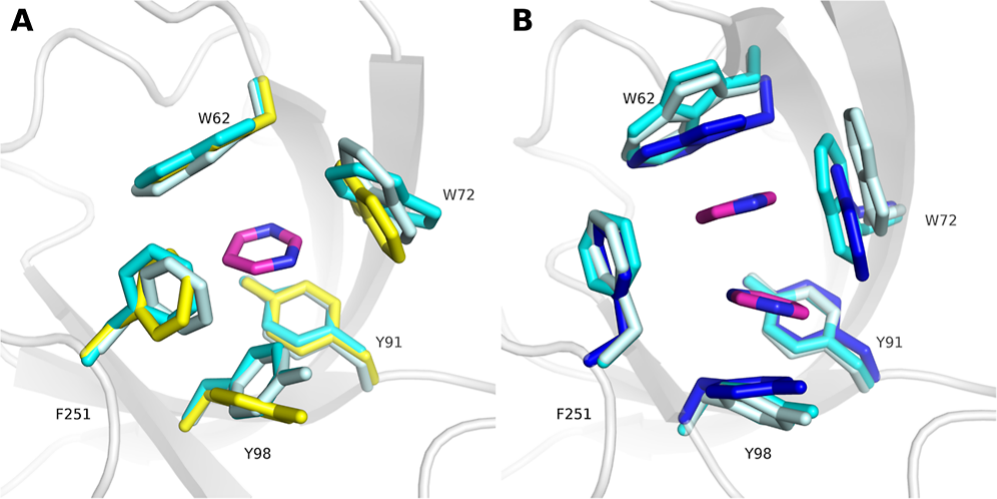
Sampled open-cage conformations (shown in different shades of cyan) with an inverted orientation of W72. Pyrimidine probe molecules are exemplarily shown with purple sticks. (A) Simulations with isoindoline (cluster 1) and pyrimidine (cluster 4) delivered conformations that are comparable to reference X-ray structure 6I8Y (yellow). (B) Cluster 2 obtained from the isoindoline simulations and cluster 5 obtained from the pyrimidine simulations show high similarity to the reference conformation found in 4H75 (dark blue).

When taking a closer look at the results obtained for the simulations using isoindoline and pyrimidine as probe molecules, we observed that some of the representative cluster structures show high pocket volumes and key distance values which indicates the presence of an open-cage structure (Table 2). However, relatively high RMSD values were calculated with respect to all open cage reference X-ray structures (PDB IDs 6I8Y, 4H75). Visual inspection of these representative cluster structures (isoindoline cluster 1, 2 and pyrimidine cluster 4, 5) revealed that the side chain of W27 adopts an inverted orientation (Figure 8A,B) thereby resulting in an elevated RMSD value. However, the cage is still present in an open-cage conformation.

The results derived from the cosolvent MD simulations in SPIN1 domain 1 do not indicate any superiority of one probe molecule in inducing an open-cage conformation. This suggests that a certain hydrophobicity of the probe molecules is sufficient in order to observe a binding event. It can be noticed, that neutral probe molecules tended to accumulate in the binding pocket (Figures 8B and S5D); that is, several molecules could bind simultaneously into the pocket, which led to the expansion of the binding pocket and the detection of higher pocket volumes (Table 2). Remarkably, only triethylamine, isoindoline, and pyrimidine were able to produce the conformation of F251 observable in the reference X-ray structure 6I8Y. These bulky or aromatic probes get completely engulfed by the surrounding aromatic residues and are stabilized by cation–pi interactions, hydrophobic interactions and/or pi–pi interactions. Simulations using triethylamine and isoindoline show high cluster fractions where F251 adopts the conformation found in 6I8Y (Table 2) emphasizing the advantage of the given conformation of F251 in minimizing the solvent exposure of bulky probe molecules (Figure S5A,B). In contrast, all cosolvents except trimethylamine induced the second open-cage conformation observable in PDB structure 4H75. In this alternative conformation, F251 shows a flipped orientation and benefits from pi–pi interactions with W62. Pyrrolidine and acetonitrile are noteworthy in this case since they only induced this particular pocket conformation. It was observed that these rather small cosolvents preferentially occupied the binding site in a position directly next to W62 due to the following reasons. First, positively charged probe molecules like pyrrolidine are attracted by E64 which is located adjacent to W62 (Figure S5C). Second, the probe molecules tend to reduce their solvent exposure by moving their hydrophobic molecule parts toward the buried spot at the bottom of W62 (Figure S5D). Simultaneously, F251 follows the movement of the cosolvents and flips to the conformation found in 4H75 in order to preserve the hydrophobic contacts or pi-cation interactions. As a small aromatic probe molecule, pyrimidine was able to significantly stabilize both open-cage conformations by adopting different orientations within the binding pocket and thus participating in distinct pi–pi interaction networks involving the surrounding aromatic residues (Figure 8A,B). In conclusion, the cage conformation found in 4H75 could be universally induced by any probe molecule with hydrophobic properties, while the pocket conformation found in 6I8Y was preferentially observed using bulky probe molecules.

SPIN1 domain 1 is known to bind a variety of ligands, including methylated arginine and lysine moieties, as well as neutral fragments (chemical additives) or bivalent inhibitors. The conformational space of the binding site in ligand-bound states appears rather limited according to the available crystal structures (Figure 4A). Therefore, it seems plausible that binding events were observable for the majority of the used probe molecules. The pocket conformation found in reference X-ray structure 4H75 was uniquely solved in the presence of the chemical additive CHES (*N*-cyclohexyl-2-aminoethanesulfonic acid) that was found to bind the aromatic pocket in SPIN1 domain 1. In the respective PDB structure, two alternative positions were stored for the cocrystallized fragment suggesting a certain flexibility within the pocket. This finding emphasizes the above-mentioned hypothesis that binders that do not exploit and stabilize the shape of the binding site observable in PDB structure 6I8Y (like the small cosolvents) are likely to promote the flipped conformation of F251 found in reference X-ray structure 4H75.

### SPIN1 Domain 2

**Figure 9:**
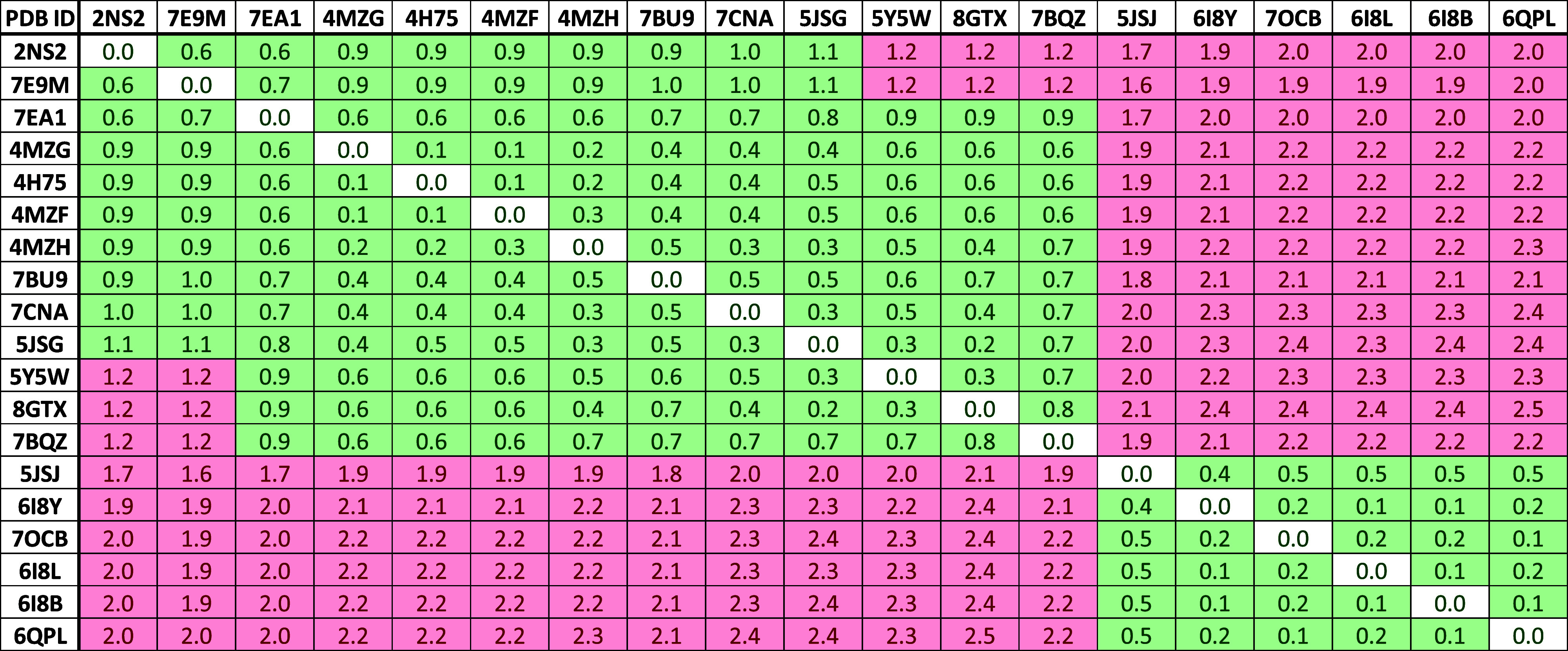
RMSD matrix of available SPIN1 crystal structures. The values were calculated for the aromatic cage residues of domain 2. A threshold of 1.2 Å was applied to highlight clusters of similar cage conformations.

Domain 2 of SPIN1 also features a (druggable) aromatic pocket for which different conformations have been described. The aromatic cage consists of four amino acid residues, namely F141, W151, Y170, and Y177. Structural superposition of all available crystal structures followed by calculation of the RMSD values for the aforementioned cage residues revealed two major clusters as shown in the RMSD matrix (Figure 9). The first cluster shows a closed state of the aromatic pocket (Figure 4B) which was observed in the apo protein structure (PDB ID 2NS2) as well as structures cocrystallized with some inhibitors or peptides featuring methylated lysines. Therefore, it can be argued that the closed conformation of this pocket is ligandable, in contrast to the closed state of domain 1. The second cluster visible in the RMSD matrix consists of structures with open aromatic pockets, characterized by a flipped conformation of F141 (Figure 4B) which allows the binding of several mono- and bivalent inhibitors.^13,34,35^ PDB structure 6I8Y was chosen as the open-cage reference.

While inspecting the selected reference X-ray structures, we noticed that one can differentiate between the open and closed states of the pocket by measuring the distance between F141-C_Z_ and Y177-C_G_. The closed form of the apo protein shows a distance value of 6.7 Å (PDB ID 2NS2), while a distance of 10.5 Å was measured for the open state (PDB ID 6I8Y) (Table 1). We tracked the distances throughout the MD simulations (Figure 6B) and noticed that in the case of the pyrrolidine simulations, the values move in a rather narrow window. The kernel density estimator shows a peak at the distance value measured for the closed conformation. Similar peaks in the distance distributions are observable for the other cosolvents. However, the latter also induced higher distance values, suggesting the sampling of the open conformation. In the case of pure water simulations, we can observe another distance peak at 5 Å, which could be interpreted as a third, collapsed conformation of the pocket.

A residual pocket volume of 183 Å^3^ was measured for the closed-cage structure (PDB ID 2NS2), whereas the open-cage structure (PDB ID 6I8Y) showed a significantly higher volume of 397 Å^3^ (Table 1). Again, we tracked the volume change throughout every MD approach. The resulting pocket volume distributions (Figure 6B) give some insight which supports the above-mentioned findings. In all the simulations, a prominent peak is visible around 180 Å^3^ suggesting high populations of the ligandable closed cage state similar to the one observed in PDB structure 2NS2. Especially in simulations using triethylamine, isoindoline and pyrimidine as probe molecules, a significant amount of frames showing pocket volumes > 397 Å^3^ can be detected, indicating that an open cage was formed.

It is worth noting, that especially in the classical simulations using only water as solvent and to a lesser extent when using the cosolvents isoindoline, acetonitrile and pyrimidine, an additional state could be observed where the pocket shows a volume of 0 Å^3^. This state represents a “collapsed”-cage conformation where the side chain of F141 flips inward to undergo face-to-face pi–pi interactions with W151, thereby leading to the collapse of the aromatic cage structure. A similar observation was made in our previous publication.^37^ Interestingly, the small charged probe molecules triethylamine, trimethylamine, and pyrrolidine seem to majorly prevent such collapse of the aromatic cage conformation as evident by the absence of frames showing pocket volumes of 0 Å^3^.

Similar to our previously described approach, all trajectories were clustered based on the heavy-atom RMSD of the aromatic cage residues’ side chains (F141, W151, Y170, Y177). The obtained representative cluster structures were further analyzed by calculating the RMSD with respect to the selected reference X-ray structures. Additionally, pocket volumes and key distances (F141-C_Z_–Y177-C_G_) were measured and averaged for all obtained clusters. The results are shown in Table 3.

**Table 3:**
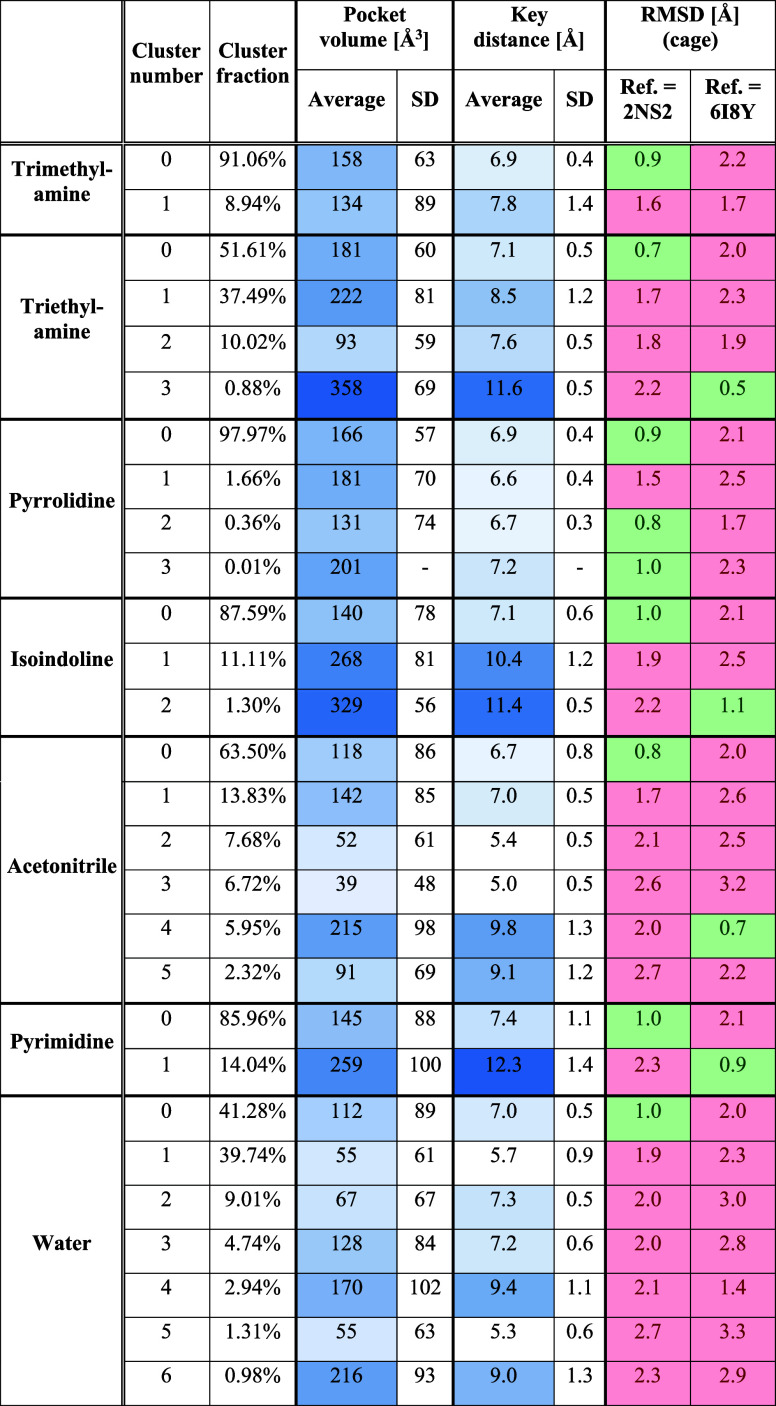
Clustering Results for the Aromatic Cage Found in SPIN1 Domain 2 and Calculated Descriptors for the Obtained Clusters (Volumes and Distances) and Representative Cluster Structures (RMSD)

**Figure 10:**
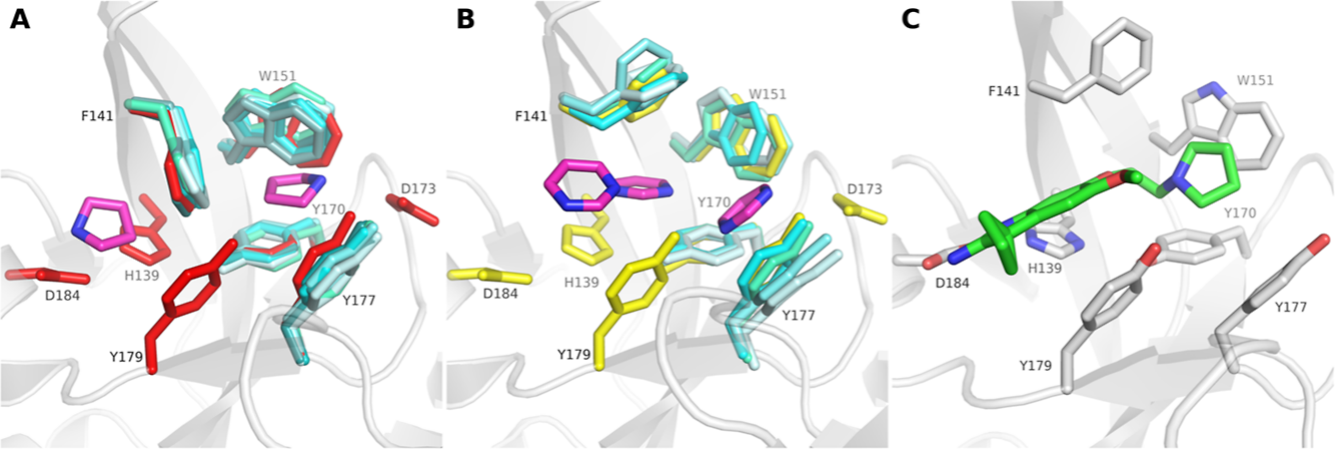
Cosolvent MDs are capable of sampling the aromatic cage conformations found in selected reference X-ray structures of SPIN1 domain 2. For the sake of clarity, the conformations of the cage-surrounding residues are only shown for the reference X-ray structures in their respective colors. (A) The closed cage conformation of 2NS2 is shown as red sticks, the highest populated cluster structures of all MD approaches reveal similar conformations (depicted in different shades of cyan; pyrrolidine probes are exemplarily shown with purple sticks). (B) The open cage state of 6I8Y is shown as yellow sticks, simulations with triethylamine (cluster 3), isoindoline (cluster 2), acetonitrile (cluster 4) and pyrimidine (cluster 1, probes exemplarily shown with purple sticks) delivered similar conformations (shown in different shades of cyan). (C) Generated docking pose for A366 (green sticks) in pyrimidine cluster 2 (white sticks).

Similar to domain 1, it is evident that the representatives of the highest-populated clusters show low RMSD values with respect to the closed-cage reference X-ray structure (Figure 10A). The simulations performed in the presence of triethylamine, isoindoline, acetonitrile and pyrimidine additionally delivered cluster structures that adopt open cage conformations as demonstrated by the low RMSD values with respect to the reference X-ray structure 6I8Y (Figure 10B). The calculated pocket volumes and distance values of the aromatic cage in these clusters show peak values when compared to other clusters obtained for the same probe molecule.

The MD trajectories were subsequently analyzed in order to investigate why the probes have different tendencies to induce the open or stabilize the closed cage conformation. Small positively charged probe molecules like trimethylamine and pyrrolidine were found to maintain the closed conformation. The inspection of respective representative cluster structures revealed that the negatively charged amino acid residues surrounding the aromatic cage might play a significant role. It could be observed that initially two probe molecules bind simultaneously; one in the closed aromatic cage and the other in an adjacent subpocket (Figure 10A). Both pockets are separated by the side chain of F141 in the closed-cage conformation. The probe molecules are extensively stabilized in the respective subpockets due to salt bridge interactions to D173 or D184 in addition to pi-cation and hydrophobic interactions to neighboring aromatic amino acid residues (Figure 10A). We could observe that this state was stabilized throughout the MD simulations using pyrrolidine and trimethylamine; hence explaining the absence of open-cage conformations during these simulations. Larger or bulkier positively charged probe molecules like triethylamine and isoindoline are less stabilized in these rather small subpockets. Visual inspection revealed that they initially bind to the aforementioned subpockets, albeit are majorly solvent exposed. The solvent-exposed probe molecules then burrow into the pocket by flipping the side chain of F141, hence forming the open-cage conformation. Neutral cosolvents like pyrimidine and acetonitrile are incapable of forming salt bridges or pi–cation interactions within the aforementioned subpockets of the closed binding site, which explains their inability to stabilize the respective conformation. By analyzing the respective trajectories, it could be noticed that these cosolvents tended to accumulate in the binding pockets. Initially, one probe molecule binds to the closed conformation while a second molecule is placed in the vicinity of F141. This triggers the flipping of F141 toward W151, resulting in its stabilization by pi–pi interactions. Meanwhile, several neutral cosolvents occupy the open binding pocket, stabilizing its conformation (Figure 10B).

SPIN1 domain 2 is known to bind a variety of ligands with its closed aromatic cage. For example, crystal structures show trimethylated lysines, unmethylated arginines, or bivalent inhibitors cocrystallized in the pocket’s closed state. Hence, it was not surprising that all probe molecules were able to bind to the closed conformation and preserve it to some extent. In contrast, the open conformation was only observed in crystallographic studies involving the presence of small-molecule inhibitors, which apparently do not fit into the closed state of the pocket. Accordingly, only probe molecules mimicking inhibitor characteristics [e.g., regarding hydrophobicity and size (also via accumulation)] or being unable to stabilize the closed state (e.g., due to the lack of a positive charge) were successful in sampling the open conformation.

Subsequently, we performed a docking study in order to investigate whether the identified open-cage cluster structures are suitable for reproducing the binding mode of the inhibitor A366. The latter binds to the open aromatic cage conformation and forms pi-cation interactions with the respective aromatic residues (F141, W151, Y170, Y177). Moreover, the inhibitor is stabilized by contacts to H139 and Y179 as well as a salt bridge to D184 (Figure S6A). Initially, the docking procedure was validated by redocking A366 into the original crystal structure, resulting in a docking pose that yields a low RMSD value of 0.5 Å (Figure S6A). Subsequently, A366 was docked into the representative cluster structures obtained from the trajectory clustering approach. The three top-scored poses according to the Glide Emodel descriptor were considered for further evaluation. RMSD values were calculated with respect to the cocrystallized inhibitor found in 6I8Y. The results demonstrate that the binding mode of A366 could be reproduced with an RMSD below 2 Å in all obtained representative cluster structures with open cage conformation: the lowest RMSD was achieved using pyrimidine cluster 1 (pose rank 1, RMSD: 1.2 Å, Figure 10C) followed by triethylamine cluster 3 (pose rank 1, RMSD: 1.4 Å, Figure S6B), acetonitrile cluster 4 (pose rank 2, RMSD: 1.8 Å, Figure S6D) and isoindoline cluster 2 (pose rank 3, RMSD: 1.9 Å, Figure S6C).

### SMN Tudor

**Figure 11:**
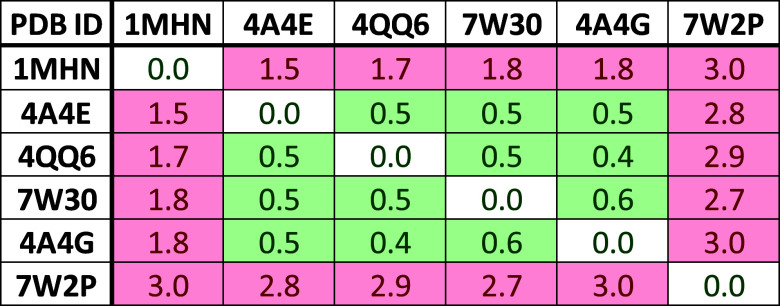
RMSD matrix of available SMN Tudor crystal structures. The values were calculated for the aromatic cage residues. A threshold of 1.2 Å was applied to highlight clusters of similar cage conformations.

The last protein that we investigated is the central Tudor domain of SMN. It features an aromatic cage consisting of four amino acids: W102, Y109, Y127, and Y130. Altogether, six protein structures are retrievable from the PDB. Calculation of the heavy-atom RMSD values for the aromatic cage residues’ side chains revealed the presence of three clusters (Figure 11). PDB ID 1MHN represents the apo protein structure and was chosen as the first reference in which a closed state of the aromatic cage is observable (Figure 4C). The other cluster contains protein–ligand complexes with bound dimethylated arginines or small molecules which are embedded in the open state of the aromatic pocket (Figure 4C); here PDB ID 4QQ6 was selected as the reference structure. Lastly, PDB ID 7W2P displays a further open-cage state with significantly different conformations of W102 and Y109 and was hence selected as a third reference structure (Figure 4C).

Since the position of W102 basically determines the ligand-accessibility to the aromatic cage, we defined the distance between W102-C_H2_ and Y130-C_G_ as an indicator for the respective states (Figure 4C and Table 1). Distance values of 4.8 (1MHN), 7.0 (4QQ6), and 7.6 Å (7W2P) were measured for the closed and the two open-cage reference structures, respectively. The results of tracking the distance values throughout the MD simulations are shown in Figure 6C. For the pure water simulations as well as simulations performed with positively charged probe molecules, a main peak is visible in the distance distributions corresponding to the closed state distance. Nevertheless, in all cases, significant shifts to higher distance values are observable as well. Particularly for the acetonitrile and pyrimidine simulations, the main distance peak corresponds to the value of the open cage reference, suggesting the sampling of the respective state.

In the apo protein structure, the aromatic pocket can be found in a collapsed state. Accordingly, we measured a pocket volume of 0 Å^3^ for 1MHN. For the open conformations found in 4QQ6 and 7W2P, pocket volume values of 157 and 211 Å^3^ were detected. Analyzing the pocket volume distributions throughout all MD simulations (Figure 6C) shows that the collapsed state of the pocket is identifiable in all applied MD approaches. Meanwhile, all herein performed cosolvent MD simulations show populations where the aromatic cage exhibits shifts to higher volume values. These populations are most predominant when acetonitrile and pyrimidine were used as probe molecules.

Trajectory clustering was carried out considering the heavy-atom coordinates of the aromatic cage residues’ side chains (W102, Y109, Y127, Y130). Subsequently, the obtained representative cluster structures were compared to the selected reference X-ray structures by calculating the RMSD values considering the same residues. Additionally, pocket volumes as well as key distance values (W102-C_H2_–Y130-C_G_) were calculated for the obtained clusters (Table 4).

**Table 4:**
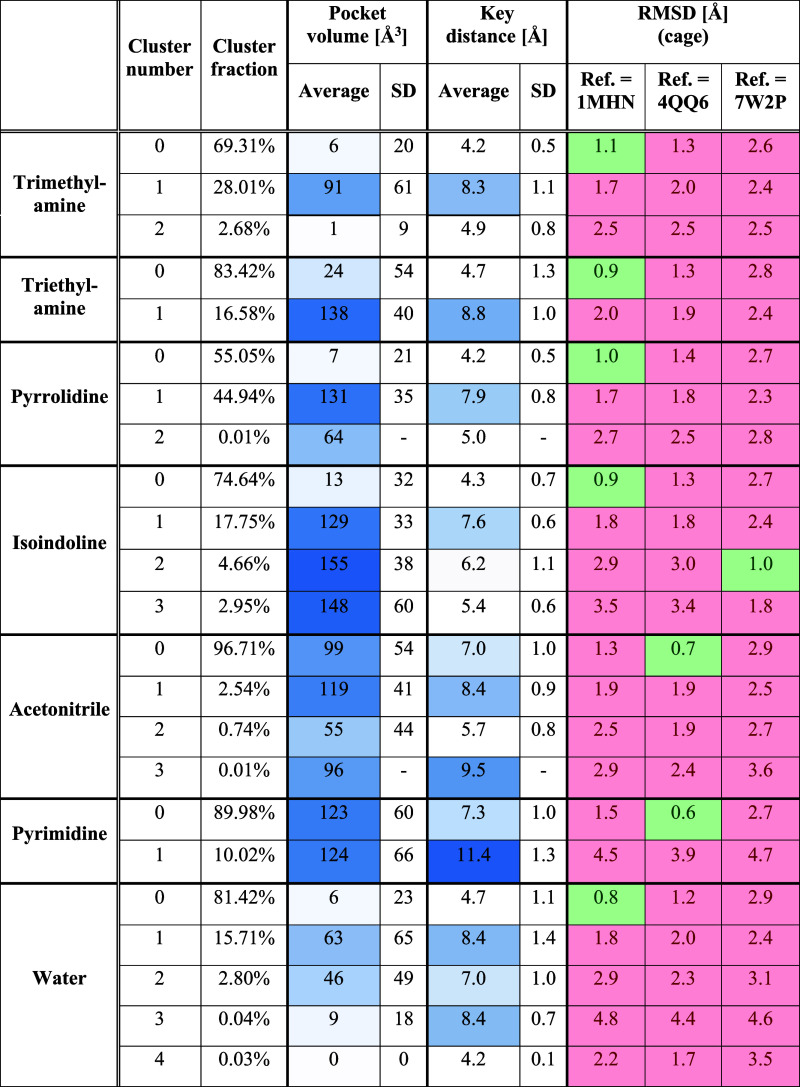
Clustering Results for the Aromatic Cage Found in the SMN Tudor Domain and Calculated Descriptors for the Obtained Clusters (Volume and Distances) and Representative Cluster Structures (RMSD)

**Figure 12:**
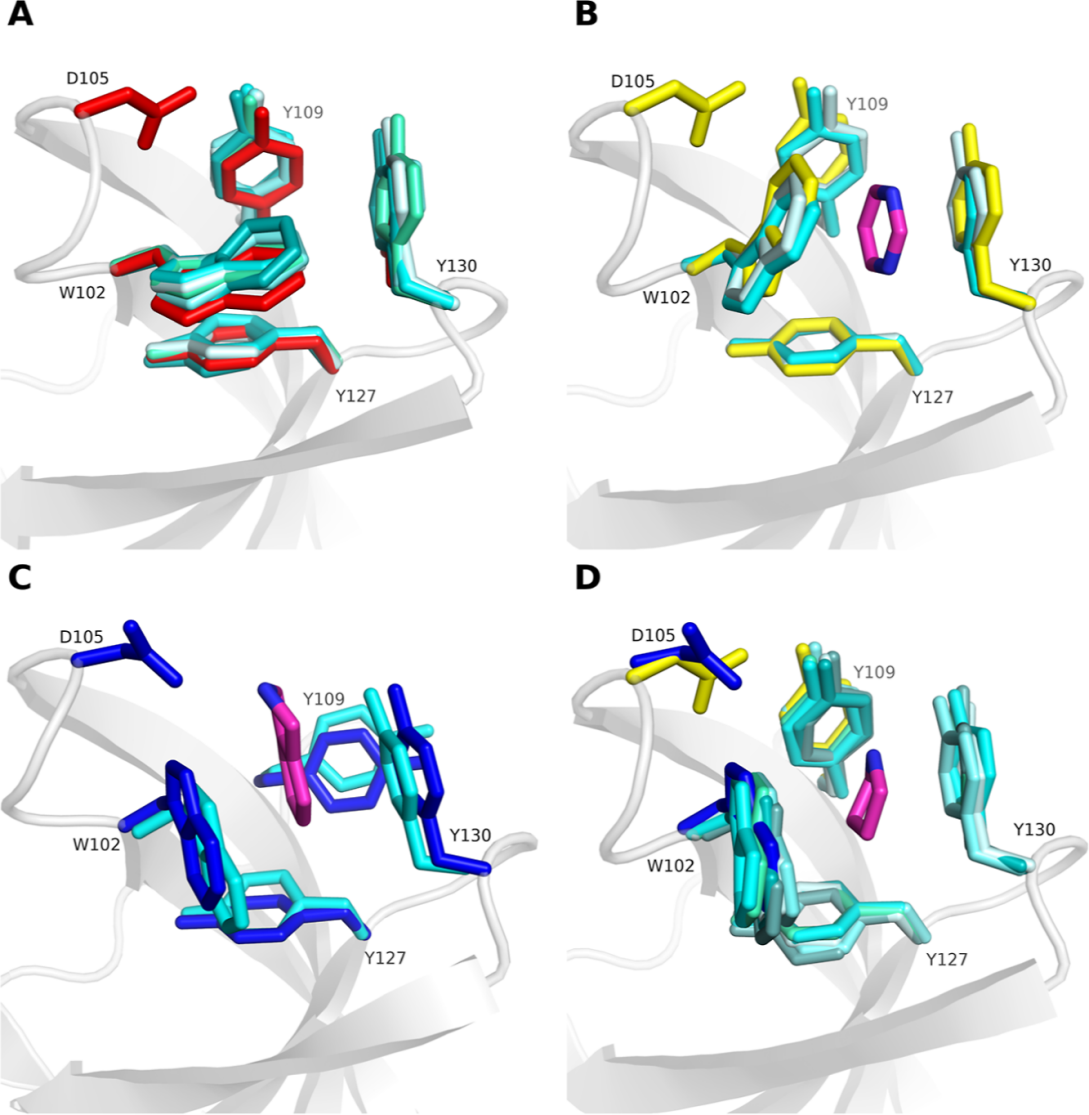
Cosolvent MDs are capable of sampling the aromatic cage conformations found in selected reference X-ray structures of the SMN Tudor domain. All representative cluster structures are depicted in different shades of cyan, while the reference X-ray structures appear in red (2NS2), yellow (4QQ6) and dark blue (7W2P). For the sake of clarity, the conformations of the adjacent residue D105 is only shown for the reference X-ray structures in their respective colors. (A) Cluster structures (clusters 0, respectively) derived from MD simulations in the presence of pure water and positively charged probe molecules show the closed conformation (2NS2). (B) Simulations with acetonitrile and pyrimidine (probe molecule exemplarily shown with purple sticks) delivered open-cage conformations similar to 4QQ6. (C) The open state found in 7W2P was only sampled by isoindoline (purple sticks) as probe molecule (cluster 2). (D) Trimethylamine, triethylamine, pyrrolidine (exemplarily shown with purple sticks), isoindoline, acetonitrile and pure water sampled conformations (clusters 1, respectively) featuring characteristics of both open-cage reference states.

Similar to the previously obtained results, almost all representatives of the highest-populated clusters reveal low RMSD values with respect to the closed cage conformation found in 1MHN (Figure 12A). In the simulations using acetonitrile and pyrimidine as cosolvents, however, the most populated clusters showed high similarity to the open conformation found in 4QQ6 (Figure 12B). Thus, a high affinity of the uncharged probe molecules to the aromatic cage can be hypothesized. Meanwhile, the open conformation of PDB ID 7W2P was only sampled by isoindoline (cluster 2, Figure 12C). Unfortunately, in this clustering approach, the calculated descriptors (pocket volume and key distance) do not clearly highlight the clusters showing open-cage conformations (Table 4) which might be explained by the high solvent exposure and therefore rather flexible nature of this binding pocket.

Visual inspection of the residual representative cluster structures revealed that the structures derived from the trimethylamine, triethylamine, pyrrolidine, isoindoline and even pure water simulations (clusters 1, respectively) feature elements of both open-cage reference X-ray structures: while Y109 adopts a conformation that is identical to 4QQ6, W102 can be found in a rotameric state that uniquely appears in 7W2P (Figure 12D). This finding explains the elevated RMSD values calculated for these structures with respect to the reference open-cage conformations, although the pocket appears in an open state. However, the available pocket volume in the cluster derived from pure water simulations is rather small compared to similar clusters obtained from cosolvent simulations (Table 4), highlighting the advantage of using cosolvents for the sampling of ligandable conformations.

**Figure 13:**
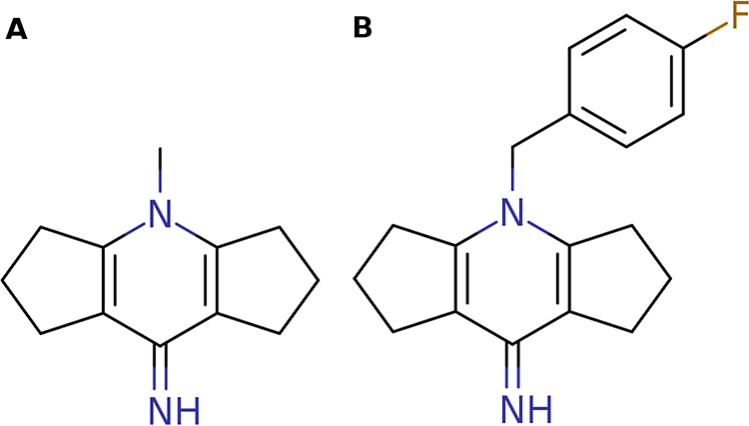
Chemical structures of SMN Tudor antagonists: compound **1** (A) and compound **4** (B).

The representative cluster structures were further analyzed in order to gain insight that could explain why the probe molecules induced different conformations. It is remarkable that all probe molecules except pyrimidine and even the pure water simulations forced W102 into the open-cage conformation only observed in X-ray structure 7W2P (Figure 12D). Visual inspection of the MD trajectories confirmed that the positively charged probe molecules are first attracted by D105 forming salt–bridge interactions and subsequently enter the pocket with their hydrophobic parts occupying the gap between W102 and Y109 (Figure 12A). In the last step, W102 shifts into a conformation where it faces Y127 showing pi–pi stacking interactions. The salt bridge between the probe molecules triethylamine and pyrrolidine and D105 is lost at the same time (Figure 12D). Conversely, the neutral probe molecules initially occupy the space between W102 and Y130 (Figure 12A) whereupon the side chain of W102 is forced to flip thereby opening the pocket to the conformation found in PDB ID 4QQ6 (Figure 12B). Furthermore, isoindoline exclusively induced the pocket residue arrangement observed in PDB ID 7W2P. As a positively charged cosolvent, it attaches to D102 with the corresponding part of the molecule, while it forms pi–pi-interactions with W102 and Y130 with the aromatic part of the molecule (Figure 12C). This interaction pattern is unique among all cosolvents and forces the probe molecule into a position close to Y109. In this position, isoindoline is capable of permanently disrupting the hydrogen bond between the phenolic hydroxyl group of Y109 and the carboxylate group of D105. The loss of the aforementioned hydrogen bond together with the larger volume of the probe molecule finally triggers the conformational change of Y109. The latter is stabilized afterward by T-shaped pi-pi-stacking interactions with Y130 (Figure 12C). In conclusion, isoindoline mimics compound **4** (Figures 13B and S7B) by offering similar interaction features and claiming additional space in the pocket, which led to the induction of the pocket conformation observed in PDB ID 7W2P.

**Figure 14:**
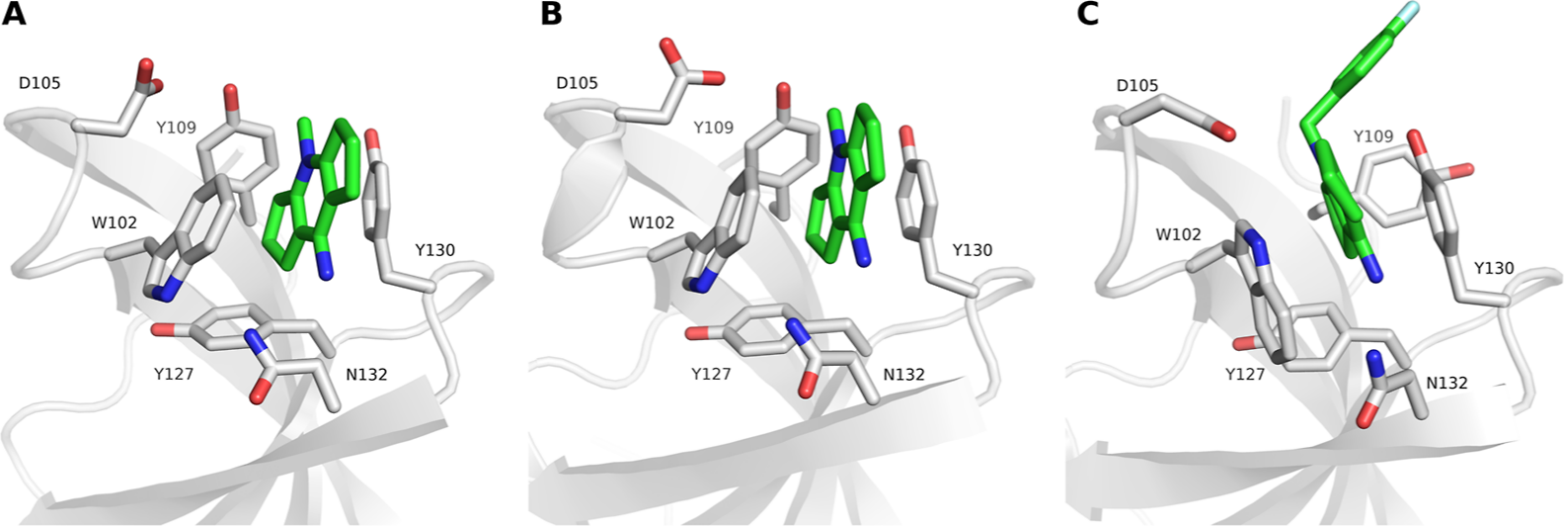
Generated docking poses (green sticks) for compound **1** in acetonitrile cluster 0 (A) and in pyrimidine cluster 0 (B) as well as for compound **4** in isoindoline cluster 2 (C). The cage residues of the representative cluster structures are shown with white sticks, respectively.

In the follow-up docking study, we investigated whether the binding modes of compounds **1** (Figure 13A) and **4** (Figure 13B) which were cocrystallized in the reference structures (PDB IDs 4QQ6 and 7W2P) could be reproduced using the obtained cluster structures. The experimentally determined binding mode of compound **1** (PDB ID 4QQ6) shows that the tricyclic scaffold is sandwiched between W102 and Y130 while forming pi–pi interactions (Figure S7A). Compound **4** (PDB ID 7W2P) shows similar receptor interactions. However, the deviating conformation of Y109 leaves more space for the additional fluoro-phenyl group (Figure S7B). Initially, we validated the docking protocol by redocking trials. For that, compound **1** was docked into its original structure 4QQ6 resulting in a docking pose showing a low RMSD value of 0.3 Å (Figure S7A). Similarly, compound **4** was docked into structure 7W2P. The docking protocol also performed well in this case, delivering a docking pose with an RMSD value of 0.6 Å (Figure S7B). Subsequently, both compounds were docked into the previously identified representative cluster structures showing the sampled reference conformations. The docking poses were selected in the same way as described for the docking study in SPIN1 domain 2. The binding mode of compound **1** could be successfully reproduced when using the highest-populated cluster structures derived from the acetonitrile (pose rank 1, RMSD: 0.7 Å, Figure 14A) and pyrimidine (pose rank 1, RMSD: 0.8 Å, Figure 14B) simulations. Furthermore, cluster 2 obtained from the isoindoline simulations allowed the reproduction of the binding mode of compound **4** (pose rank 3, RMSD: 1.4 Å, Figure 14C).

## Conclusions

In this study, we aimed to assess whether cosolvent MD simulations are suitable tools to sample apo and inhibitor-bound aromatic cage conformations that have been observed in crystallographic studies. In all studied cases, we could show that the different pocket conformations observed in the X-ray structures could be reproduced. Trajectory clustering turned out to be a helpful tool for identifying representative conformations that occur during the MD simulations. We could show that the tracking of predefined distances between key binding site residues as well as changing pocket volumes during the simulations can assist in identifying different conformational states. Moreover, the same descriptors calculated for representative cluster structures are helpful indicators for recognizing structures featuring open cage conformations. However, the limitations of these descriptors were noticeable, especially with flexible binding sites like the one found in the SMN Tudor domain. We also investigated the influence of using different probe molecules on the conformational sampling. We could not identify a single probe molecule that performed best in reproducing all reference cage conformations. We rather conclude that the optimal probe molecule choice depends on the nature of the binding site. More precisely, the current study reveals that the cage-surrounding residues and their interactions with the probe molecules additionally affect the dynamics of the binding events. The neutral probe molecules acetonitrile and pyrimidine seemed to have slight advantages in this study since these cosolvents were able to sample at least one open cage conformation in every of the presented cases. Thus, a positive charge was discovered to be unnecessary in order to observe binding events during the simulations. Instead, a certain hydrophobicity seems to be crucial for binding since protonated probe molecules like trimethylamine performed poorly in sampling open cage conformations. Anyhow, the superiority of using cosolvents for the sampling of binding site conformations was demonstrated since the pure water simulations were not able to sample any of the open cage states predefined by the reference X-ray structures. In conclusion, we suggest using at least one of the neutral probe molecules when exploring the conformational flexibility of an aromatic pocket. Additionally, a case-adapted probe molecule based on the structure of known ligands should be used in order to complete the proper sampling of potential binding site conformations. For example, a probe molecule like isoindoline with matching features compared to published antagonists (aromaticity combined with a positive charge) was necessary in the case of the SMN Tudor domain in order to sample the residual conformational state. It also showed good results in both domains of Spindlin 1; isoindoline is also found as cage-binding substructure in some Spindlin 1 inhibitors (Figure 2B). Finally, we were able to show that the representative cluster structures derived from MD simulations with cosolvents are suitable conformations for inhibitor docking, as they were found to be in reasonable agreement with the experimental binding modes of known inhibitors.

## Data Availability

All crystal structures used in the current work are retrievable from the RCSB protein data bank (https://www.rcsb.org/). The protein structures were prepared with the aid of the Protein Preparation Wizard (Schrodinger 2021–3). All MD simulations were carried out using Amber 22 software. Trajectory analysis was performed using CPPTRAJ (Amber Tools 22). Fpocket (4.0) was used to extract pocket shapes, MDpocket (4.0) executed the pocket volume calculations (https://github.com/Discngine/fpocket). Further analysis of obtained representative cluster structures was done using Schrödinger’s graphical interface Maestro (2021–3). Ligand preparation for docking implied LigPrep and ConfGen of the Schrodinger software suite (2021–3). The docking studies were carried out using Glide (2021–3). Microsoft Excel and Matplotlib were used for plot generation. Figures were created using PyMol (1.8.4.0), Marvin Sketch (19.19.0 2019) and Maestro (2021–3). The Supporting Information features Amber scripts, topologies and input coordinates that were used to generate the presented results. Additionally, the representative cluster structures as well as the docking results are provided.

## Supporting Information Available

Additional information about the built systems for MD simulation and figures visualizing pocket shapes and 2D inhibitor structures as well as further depictions of MD and docking results (PDF)
Topology and coordinate files of the solvated systems; scripts used for running the MD simulations; representative cluster structures obtained for generated MD trajectories; docking poses generated for A366 and compounds **1** and **4** (ZIP)
