# Supplementary material for: Exploring Aromatic Cage Flexibility Using Cosolvent Molecular Dynamics Simulations—An In-Silico Case Study of Tudor Domains
Source: J Chem Inf Model. 2024 May 21;64(11):4553–69. doi: 10.1021/acs.jcim.4c00298 (PMC11167732; doi:10.1021/acs.jcim.4c00298)
Supplement: Supplementary file 1 — ci4c00298_si_001.pdf [file ci4c00298_si_001.pdf]

Exploring Aromatic Cage Flexibility Using Cosolvent Molecular Dynamics Simulations – An In-Silico Case Study of Tudor Domains

*Christopher Vorreiter, Dina Robaa, Wolfgang Sippl**

Department of Medicinal Chemistry, Institute of Pharmacy,

Martin-Luther-University of Halle-Wittenberg, 06120 Halle (Saale), Germany

Email: wolfgang.sippl@pharmazie.uni-halle.de

Supporting Information

S1-S8: Molecular dynamics and docking results

Table S1. Built systems for the cosolvent MD simulations. The number of added probe and water molecules to achieve target concentration as well as the volumes of the resulting systems are listed.

System		Cosolvent molecules	Water molecules	Volume [Å^3]
SPIN1	Trimethylamine	220	12,297	525,843
	Triethylamine	221	12,284	577,966
	Pyrrolidine	222	12,188	532,074
	Isoindoline	222	12,261	555,697
	Acetonitrile	221	12,293	512,599
	Pyrimidine	224	12,371	533,091
	Pure water	0	12,295	455,412
SMN Tudor	Trimethylamine	109	6,088	253,468
	Triethylamine	110	6,086	282,067
	Pyrrolidine	109	6,080	258,274
	Isoindoline	110	6,095	268,754
	Acetonitrile	110	6,078	245,602
	Pyrimidine	109	6,077	252,188
	Pure water	0	6,093	218,385

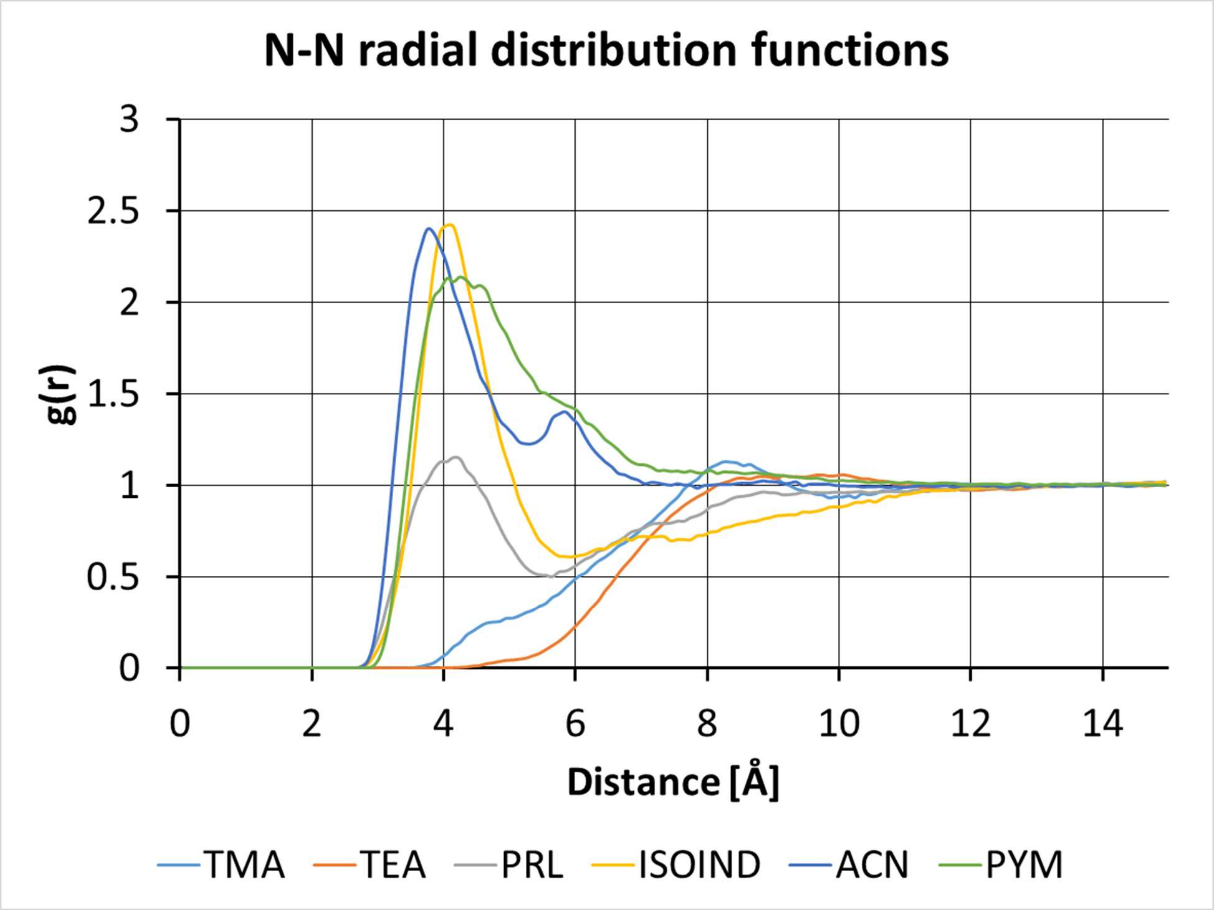

Figure S1. N-N radial distribution functions calculated for the probe molecules in respective MD simulations. TMA: trimethylamine, TEA: triethylamine, PRL: pyrrolidine, ISOIND: isoindoline, ACN: acetonitrile, PYM: pyrimidine.

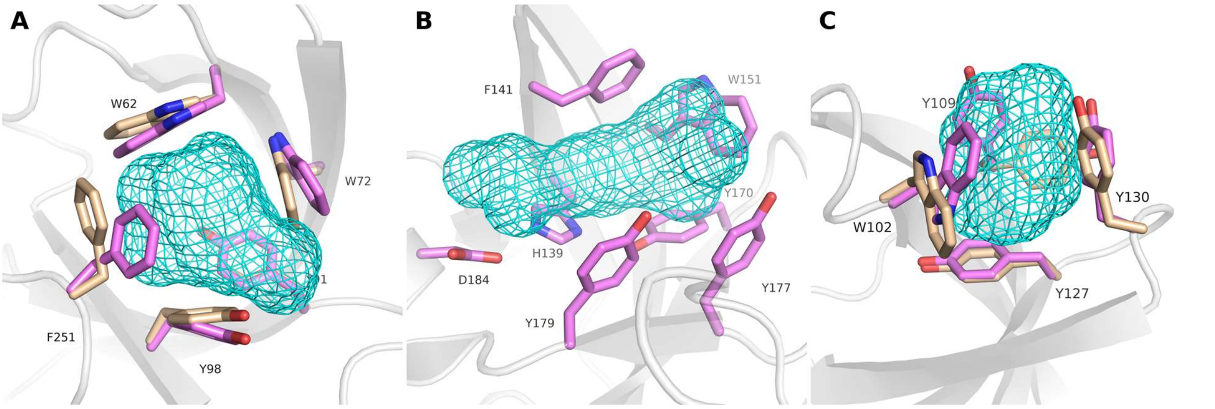

Figure S2. Pocket shapes extracted with Fpocket. **A**: SPIN1 domain1, **B**: SPIN1 domain 2, **C**: SMN Tudor domain.

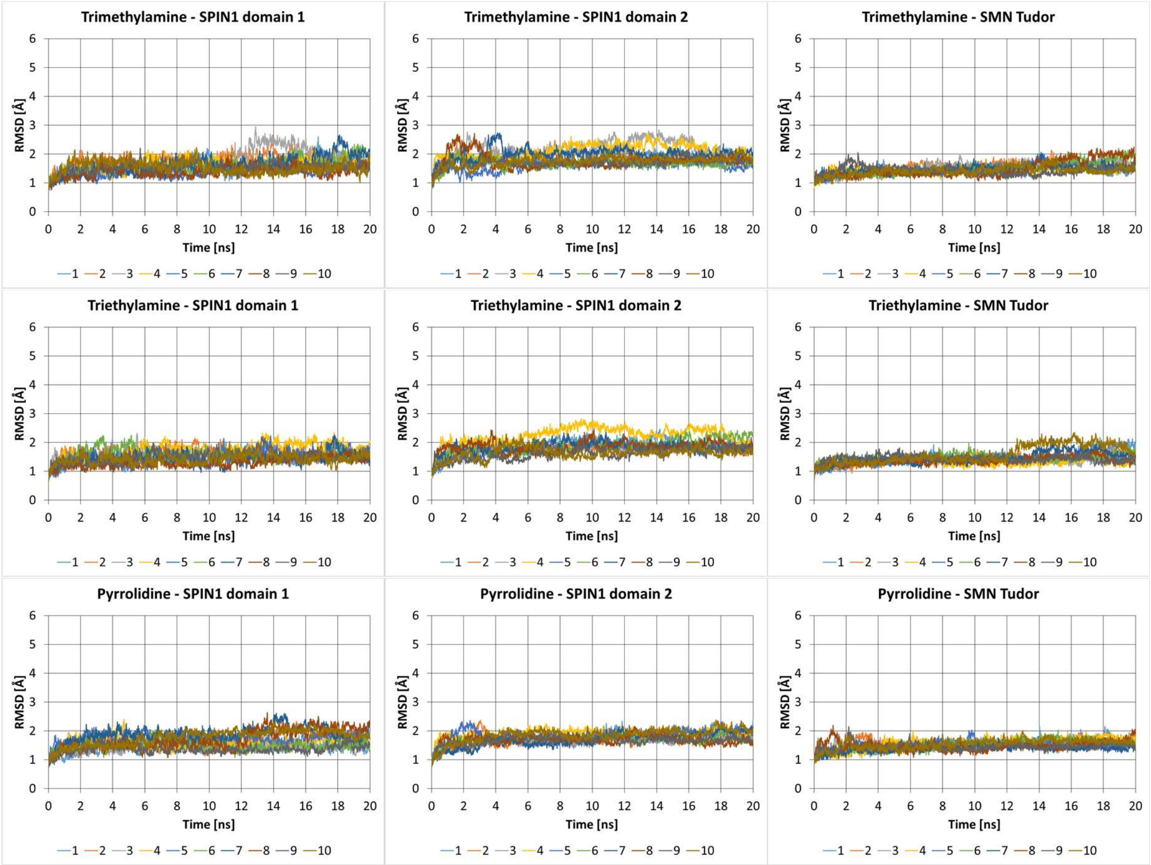

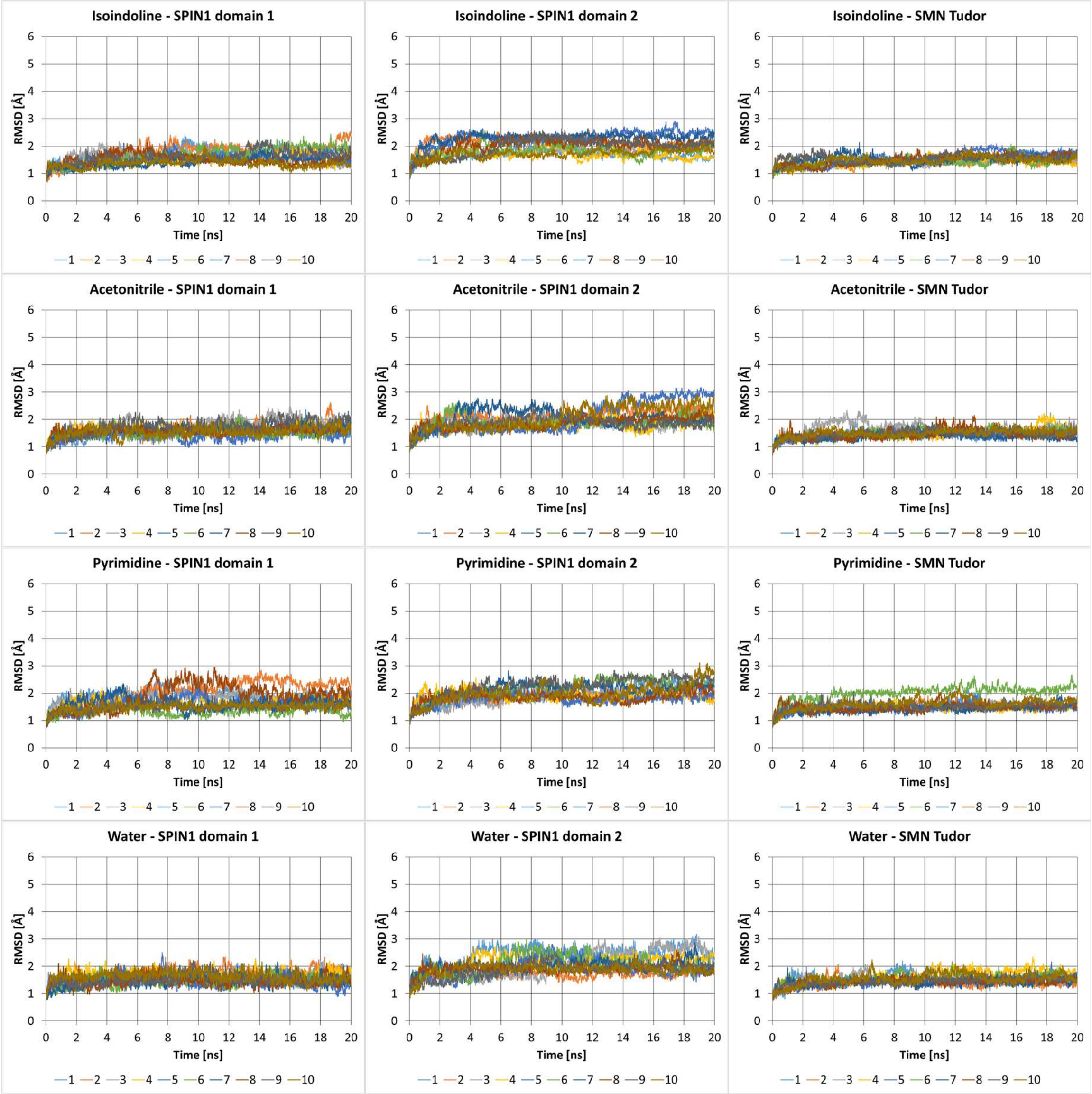

Figure S3. Plots showing the RMSD progressions during all performed MD simulations. Values were calculated considering respective proteins‘ heavy atoms. Since the termini of the SMN Tudor domain fluctuated significantly, the first and the last three amino acid residues of the protein were not considered in the RSMD calculations.

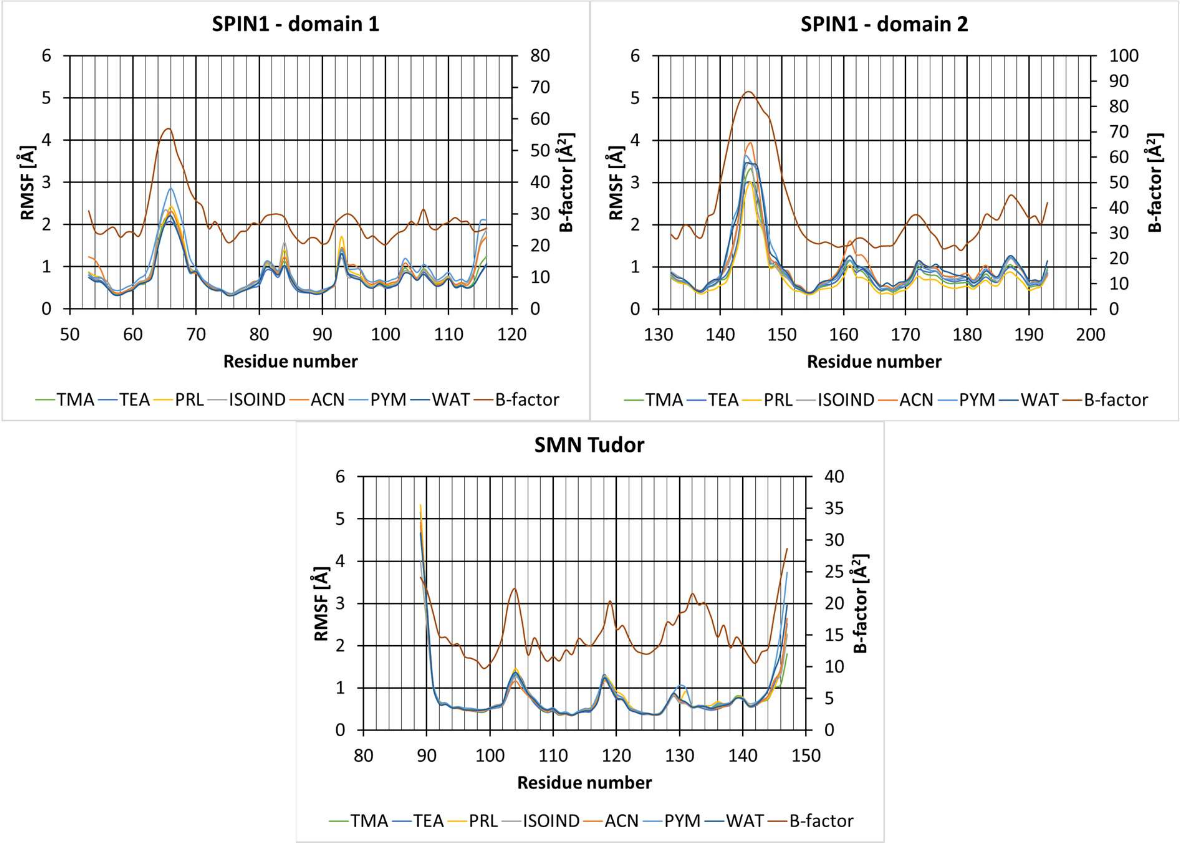

Figure S4. RMSF plots visualizing the amino acid fluctuations during all MD simulations. B-factor values of apo protein crystal structures are provided for comparison. TMA: trimethylamine, TEA: triethylamine, PRL: pyrrolidine, ISOIND: isoindoline, ACN: acetonitrile, PYM: pyrimidine.

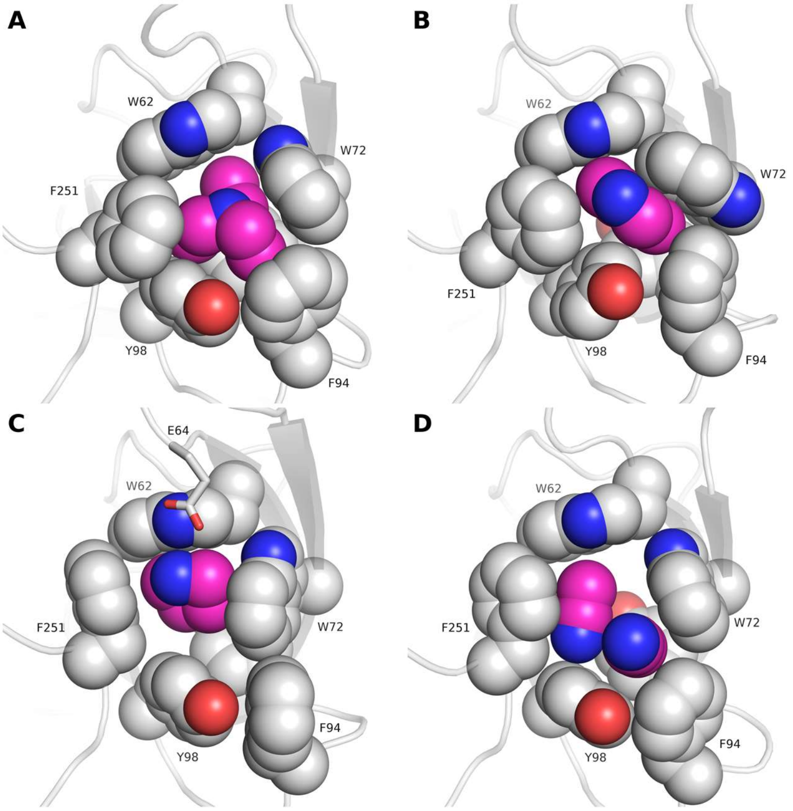

Figure S5. Representative cluster structures obtained for the aromatic cage of SPIN1 domain 1 visualizing the bound states of the probe molecules. The aromatic pocket residues are depicted with white spheres, the probe molecules appear as purple spheres. **A**: Triethylamine cluster 2, **B**: Isoindoline cluster 1, **C**: Pyrrolidine cluster 2, **D**: Acetonitrile cluster 2.

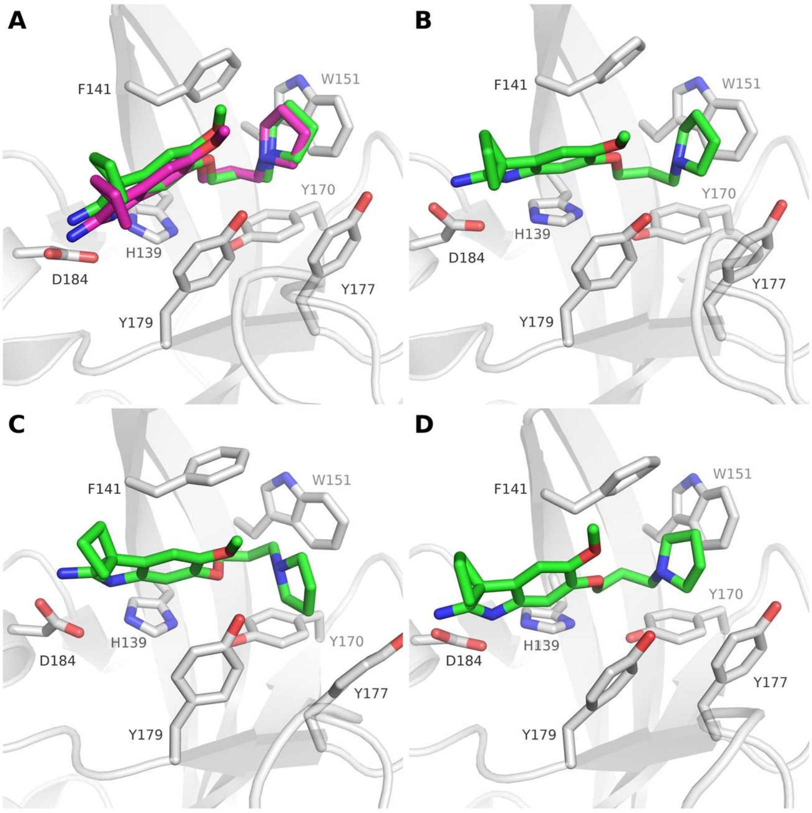

Figure S6. Generated docking poses for A366 in SPIN1 domain 2. The protein structures are shown with white sticks, the docking pose is visualized by green sticks. The reference pose found in 6I8Y is shown with purple sticks. **A**: Redocking in 6I8Y, **B**: Docking in triethylamine cluster 3, **C**: Docking in isoindoline cluster 2, **D**: Docking in acetonitrile cluster 4.

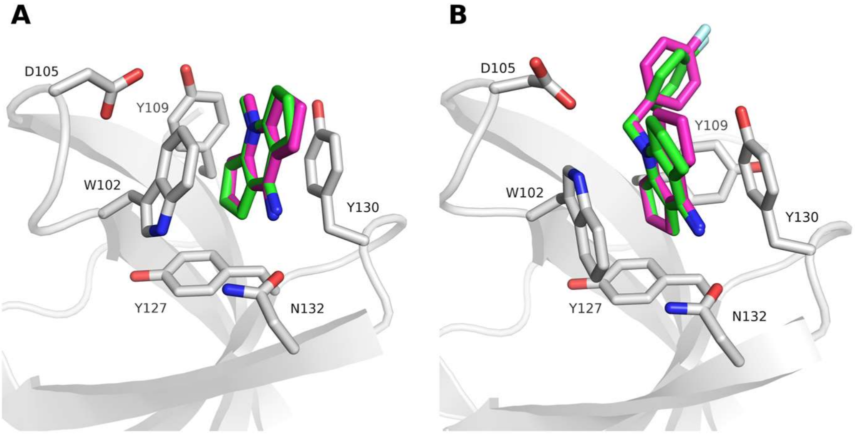

Figure S7. Redocking results for the aromatic pocket of SMN Tudor. The docking poses appear as green sticks while the reference poses are depicted with purple sticks, respectively. The aromatic cage residues are visualized with white sticks. **A**: Redocking of compound 1 into 4QQ6, **B**: Redocking of compound 4 into 7W2P.
